# Intestinal Epithelial MHC-II at the Interface of Microbiota, Immunity, and Inflammation

**DOI:** 10.3390/ijms27167271

**Published:** 2026-08-14

**Authors:** Sarah de Oliveira, José Luís Fachi

**Affiliations:** 1 Department of Genetics, Evolution, Microbiology, and Immunology, Institute of Biology, State University of Campinas, Campinas 13083-862, SP, Brazil; saa.oliveira31@gmail.com; 2 Department of Microbiology and Immunology, Virginia Commonwealth University School of Medicine, Richmond, VA 23298-0678, USA

**Keywords:** intestinal epithelial cells, MHC-II, antigen presentation, gut microbiota, mucosal barrier, T helper cells, inflammatory bowel disease

## Abstract

Major histocompatibility complex class II (MHC-II) expression by intestinal epithelial cells (IECs) has emerged as a critical mechanism regulating mucosal immune homeostasis at the interface between the intestinal microbiota, epithelial barrier, and immune system. Beyond professional antigen-presenting cells, IEC-intrinsic MHC-II shapes CD4^+^ T-cell responses, influencing tolerance to commensal microorganisms, immunity to enteric pathogens, and maintenance of barrier integrity. Recent studies have revealed that epithelial MHC-II expression is dynamically regulated by cytokines, the gut microbiota, dietary factors, and microbiota-derived metabolites, linking environmental signals to local adaptive immune responses. Dysregulation of epithelial antigen presentation has been implicated in chronic intestinal inflammation, including inflammatory bowel disease (IBD). This review summarizes current knowledge regarding the molecular regulation, spatial organization, and immunological functions of epithelial MHC-II and discusses its emerging role in host–microbiota interactions, mucosal barrier homeostasis, and intestinal disease.

## 1. Introduction

The intestinal epithelium serves as a highly specialized interface between the luminal environment and the host mucosal immune system. Every day, this single layer of epithelial cells is exposed to an extraordinary diversity of dietary antigens, commensal microorganisms, microbial metabolites, and potential pathogens while simultaneously maintaining efficient nutrient absorption and preserving tissue integrity [1,2]. Unlike most epithelial barriers, the intestinal epithelium must continuously discriminate between harmless and pathogenic stimuli, mounting protective immune responses against invading microorganisms while maintaining tolerance to food antigens and the resident microbiota. Failure to maintain this delicate balance results in chronic inflammation, barrier dysfunction, and increased susceptibility to infectious and immune-mediated diseases [3,4].

Historically, intestinal epithelial cells (IECs) were viewed primarily as structural components of the mucosal barrier. However, advances in genetic models, organoid systems, and single-cell technologies have established IECs as active regulators of mucosal immunity. Through their ability to sense microbial and dietary signals and communicate with resident immune cells, IECs coordinate immune surveillance, tissue repair, and maintenance of intestinal homeostasis [3,5].

Among the mechanisms that contribute to immune regulation at the intestinal barrier, antigen presentation through major histocompatibility complex class II (MHC-II) has emerged as one of the most intriguing and least understood. Antigen presentation is traditionally considered a specialized function of professional antigen-presenting cells (APCs), including dendritic cells, macrophages, and B cells, which initiate and shape adaptive immune responses. However, both human and murine studies have demonstrated that intestinal epithelial cells (IECs) express MHC-II molecules together with components of the antigen-processing machinery, enabling them to present peptide antigens to CD4^+^ T cells [6]. Given their unique anatomical position at the interface between luminal contents and the mucosal immune system, IECs are ideally positioned to integrate environmental signals and influence local adaptive immune responses. Emerging evidence suggests that epithelial MHC-II contributes to the regulation of host–microbiota interactions, maintenance of mucosal homeostasis, and coordination of immune responses during infection and inflammation [7,8,9,10,11]. These observations raise a fundamental biological question: why would a highly specialized barrier cell evolve the capacity to present antigen?

For many years, epithelial MHC-II was largely viewed as a byproduct of inflammation. Because MHC-II is strongly induced by interferon-γ (IFNγ), increased epithelial expression observed in inflammatory bowel disease (IBD) [12], celiac disease [13], graft-versus-host disease (GVHD) [7], and enteric infections [14] was generally interpreted as a consequence of local inflammatory cytokine production rather than a mechanism with intrinsic physiological significance. Consequently, the functional relevance of epithelial MHC-II remained controversial. Early studies produced conflicting results, suggesting that IECs could either promote effector T-cell activation [6] or induce immune tolerance through regulatory T cells [8], while uncertainty regarding the expression of co-stimulatory molecules further complicated the interpretation of epithelial antigen presentation.

Recent technological and conceptual advances have fundamentally reshaped this view. Conditional genetic models have demonstrated that epithelial MHC-II directly influences intestinal immune responses in vivo [7,9], while organoid systems have enabled mechanistic studies of epithelial antigen presentation independent of professional APCs [8]. At the same time, single-cell sequencing has revealed remarkable heterogeneity among intestinal epithelial subsets, identifying region-specific and lineage-specific differences in MHC-II expression along the crypt–villus and proximal–distal axes of the intestine [7,10]. These findings suggest that epithelial antigen presentation is not a uniform property of all IECs but rather a highly regulated program influenced by epithelial differentiation, anatomical location, and local environmental cues.

Equally important has been the recognition that epithelial MHC-II integrates signals derived from the intestinal microenvironment. The commensal microbiota has emerged as a central regulator of epithelial MHC-II expression, acting through cytokine networks and innate immune pathways to shape antigen presentation under homeostatic conditions [10,11]. More recently, dietary factors and microbiota-derived metabolites have also been shown to influence epithelial MHC-II expression [11,14], linking nutritional status and microbial metabolism to adaptive immune regulation at the epithelial barrier. Together, these discoveries support a model in which epithelial MHC-II functions as an environmental sensor that integrates microbial, dietary, and inflammatory signals to fine-tune local CD4^+^ T-cell responses.

These advances have important implications for intestinal disease. Dysregulated epithelial MHC-II expression has been implicated in chronic inflammatory disorders, including IBD [12] and celiac disease [13], as well as in graft-versus-host disease [7] and enteric infections [14]. However, accumulating evidence indicates that epithelial MHC-II cannot be simply classified as either protective or pathogenic. Instead, its biological consequences appear to depend on multiple contextual factors, including the anatomical location within the intestine, the nature of the antigen presented, the inflammatory milieu, the composition of the microbiota, and the responding T-cell population. Defining these context-dependent functions has therefore become one of the central challenges in understanding epithelial immune regulation.

In this review, we synthesize recent advances in our understanding of MHC-II expression by intestinal epithelial cells, with particular emphasis on the molecular mechanisms regulating epithelial antigen presentation, the regional specialization of MHC-II expression along the intestinal tract, and the emerging roles of epithelial MHC-II in orchestrating host–microbiota interactions and CD4^+^ T-cell responses. We also discuss the growing evidence linking epithelial MHC-II to intestinal inflammation and barrier-associated immune homeostasis and highlight key unresolved questions that will shape future investigations and therapeutic strategies targeting epithelial–immune crosstalk.

## 2. The Intestinal Epithelium as an Immune Interface

**Figure 1:**
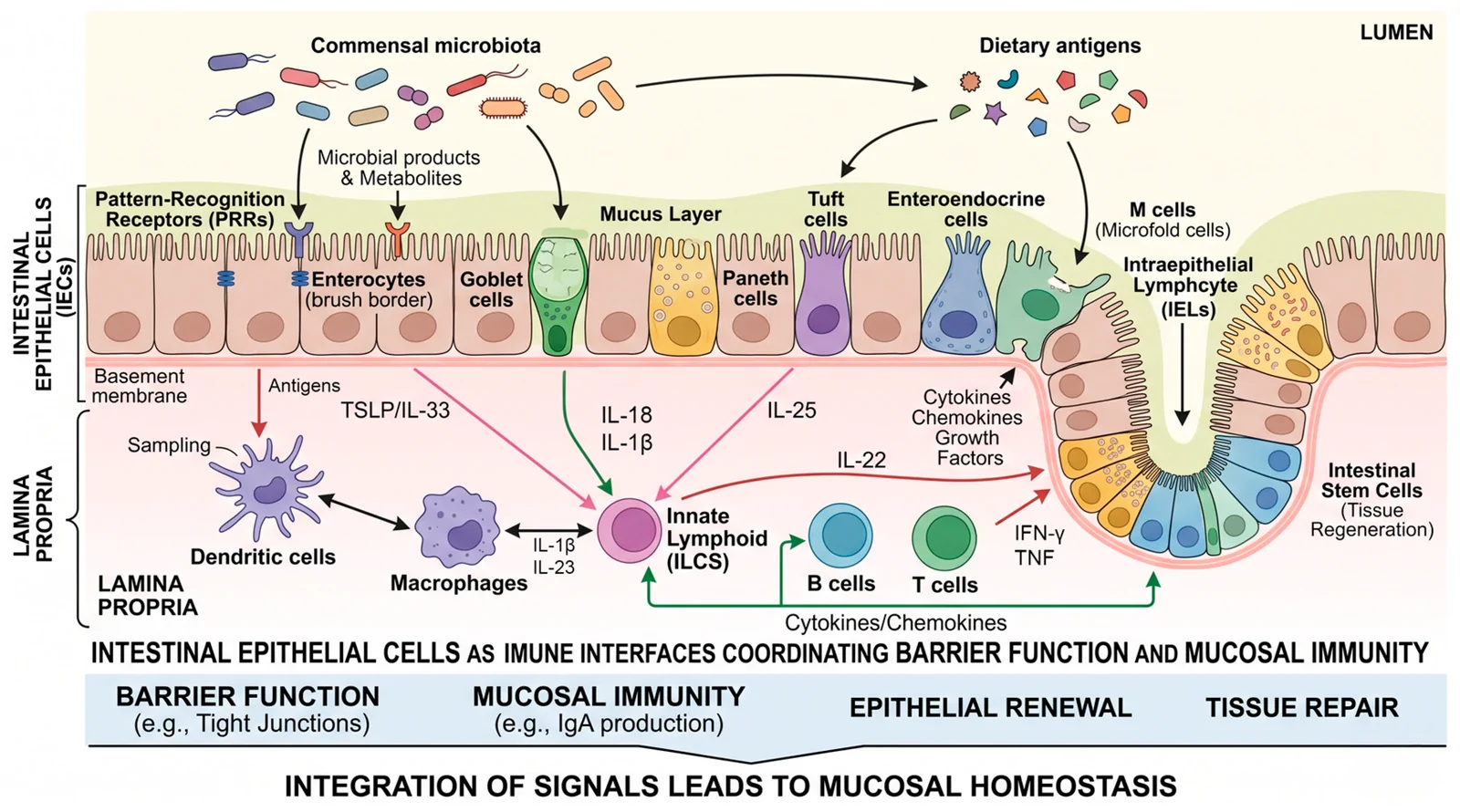
Intestinal epithelial cells as immune interfaces coordinating barrier function and mucosal immunity. The intestinal epithelium functions as a dynamic interface between the luminal environment and the host immune system. Distinct epithelial populations—including enterocytes, goblet cells, Paneth cells, tuft cells, enteroendocrine cells, microfold (M) cells, and intestinal stem cells—perform specialized roles in nutrient absorption, barrier maintenance, antimicrobial defense, antigen sampling, and tissue regeneration. Intestinal epithelial cells (IECs) continuously integrate signals derived from dietary antigens, commensal microorganisms, microbial metabolites, and pathogens through pattern-recognition receptors and other environmental sensing pathways. Bidirectional communication with intraepithelial lymphocytes (IELs), innate lymphoid cells (ILCs), dendritic cells, macrophages, and adaptive lymphocytes coordinates epithelial renewal, immune surveillance, and tissue repair. Together, these interactions establish the intestinal epithelium as an active regulator of mucosal homeostasis rather than a passive physical barrier.

The intestinal epithelium represents one of the most specialized barrier tissues in the body, functioning at the intersection of nutrient absorption, microbial containment, and immune regulation [15,16]. Unlike most epithelial surfaces, which primarily separate host tissues from the external environment, the intestinal epithelium is continuously exposed to an immense and highly diverse antigenic burden that includes dietary proteins, commensal microorganisms, microbial metabolites, environmental antigens, and enteric pathogens [1]. Despite this constant stimulation, inflammation is not the physiological default. Instead, the healthy intestine maintains a finely balanced state in which protective immunity against pathogens coexists with immunological tolerance toward beneficial microbes and innocuous dietary antigens. Preserving this equilibrium requires continuous communication between epithelial cells and the underlying immune system, positioning intestinal epithelial cells (IECs) as active regulators of mucosal immunity rather than passive structural barriers (Figure 1).

The intestinal epithelium is organized into anatomically and functionally distinct compartments that shape regional immune responses [17]. Throughout the intestine, epithelial renewal is sustained by Lgr5^+^ intestinal stem cells residing at the base of the crypts, which continuously generate progenitor cells that differentiate into mature epithelial lineages while migrating toward the luminal surface. This rapid turnover, completed within only a few days under homeostatic conditions, enables the epithelium to maintain barrier integrity despite constant exposure to mechanical stress and microbial challenge [18,19,20]. However, epithelial organization differs markedly between the small intestine and the colon. The small intestine contains crypt–villus units that maximize nutrient digestion and absorption, whereas the colon lacks villi and instead consists of deep crypts opening onto a flat epithelial surface optimized for water absorption and maintenance of host–microbiota segregation [16]. These structural differences are accompanied by profound regional variation in microbial density, metabolite availability, mucus organization, and immune cell composition, collectively creating distinct immunological niches along the gastrointestinal tract.

Recent advances in single-cell transcriptomics have further transformed our understanding of epithelial organization by revealing remarkable heterogeneity among IEC populations. Rather than representing a uniform barrier, the intestinal epithelium consists of multiple specialized cell lineages with distinct developmental trajectories and immune functions. Enterocytes, the predominant epithelial population, not only mediate nutrient absorption but also respond dynamically to microbial and inflammatory stimuli through the production of cytokines, chemokines, antimicrobial proteins, and metabolic mediators [5,17]. Goblet cells synthesize the gel-forming mucin MUC2, generating the mucus layer that physically separates luminal microorganisms from the epithelial surface, particularly in the colon [21,22,23]. Paneth cells, located at the base of small intestinal crypts, secrete antimicrobial peptides including α-defensins and lysozyme while simultaneously supporting the intestinal stem cell niche through the production of Wnt ligands and other trophic factors [24,25]. In contrast, the colonic crypt base contains deep crypt secretory cells, a Reg4-expressing epithelial population that performs analogous niche-supporting functions despite differing developmental origins [26,27]. Additional epithelial subsets, including enteroendocrine cells, tuft cells, and microfold (M) cells, contribute to luminal sensing [28,29], type 2 immunity [30], and antigen transport [31], respectively. Collectively, these observations have established that epithelial specialization extends far beyond nutrient absorption, encompassing diverse functions in tissue maintenance, microbial surveillance, and immune regulation.

The intimate relationship between the intestinal epithelium and the resident microbiota has emerged as a defining feature of mucosal homeostasis. Commensal microorganisms continuously influence epithelial physiology through the production of metabolites, microbial-associated molecular patterns, and other bioactive molecules that regulate epithelial differentiation, metabolism, and barrier function. Short-chain fatty acids generated by bacterial fermentation reinforce epithelial integrity by promoting tight junction assembly, cellular metabolism, and anti-inflammatory signaling [32,33], whereas tryptophan metabolites, secondary bile acids, and lactate influence epithelial renewal and immune responses through distinct metabolic pathways [3,34,35]. Conversely, IECs actively shape microbial ecology by producing mucus, antimicrobial peptides, secretory immunoglobulin transport machinery, and nutritional substrates that determine microbial spatial organization and community composition. Rather than functioning as independent entities, the epithelium and microbiota therefore constitute an integrated ecological unit whose reciprocal interactions maintain tissue homeostasis and confer resilience against infection and inflammatory disease.

Central to these interactions is the ability of IECs to sense changes in their luminal environment and translate them into coordinated immune responses. IECs express an extensive repertoire of pattern-recognition receptors, including Toll-like receptors [36,37], NOD-like receptors [38,39] and inflammasome-associated sensors that detect microbial- and damage-associated molecular patterns [40]. Importantly, epithelial responsiveness is tightly regulated both spatially and temporally. Differential receptor expression along the crypt–villus axis, polarized receptor localization, and multiple intracellular regulatory mechanisms allow epithelial cells to respond appropriately to invading pathogens while avoiding chronic activation by the commensal microbiota [41]. Activation of these sensing pathways induces the secretion of antimicrobial peptides, cytokines, chemokines, alarmins, and growth factors that orchestrate local immune responses while preserving epithelial integrity. Consequently, the intestinal epithelium functions as a highly specialized sensory platform that continuously integrates microbial, dietary, and inflammatory information from the intestinal lumen.

These epithelial-derived signals establish extensive bidirectional communication with resident immune cells. Intraepithelial lymphocytes (IELs), positioned within the epithelial layer itself, provide rapid immune surveillance by eliminating infected or damaged epithelial cells while contributing to tissue repair and barrier maintenance [42,43]. Their development and survival are critically dependent upon epithelial-derived signals, particularly IL-15 [44,45]. Beneath the epithelial layer, IECs further regulate the activity of dendritic cells, macrophages, innate lymphoid cells (ILCs), neutrophils, and adaptive lymphocytes through the secretion of cytokines and chemokines including thymic stromal lymphopoietin (TSLP) [46], transforming growth factor-β (TGF-β [47], IL-18 [48], IL-25 [30,49], and CCL25 [50]. In turn, immune-derived cytokines such as IFNγ, IL-22, IL-17, TNF, and IL-13 profoundly influence epithelial proliferation [17,51], antimicrobial defense [52], mucus production [53], and barrier repair [54]. This reciprocal dialogue enables the epithelium to function as both an effector and a target of immune regulation, ensuring that tissue responses remain appropriately adapted to environmental challenges.

Among the many mechanisms through which IECs communicate with the immune system, antigen presentation has emerged as one of the most unexpected and conceptually significant. Although professional antigen-presenting cells remain essential for the priming of naïve T cells within organized lymphoid tissues, increasing evidence demonstrates that IECs express major histocompatibility complex class II (MHC-II) molecules together with components of the antigen-processing machinery [5]. Unlike dendritic cells or macrophages, epithelial cells are strategically positioned at the immediate interface between luminal antigens and the mucosal immune system, allowing them to directly integrate environmental signals with local adaptive immune responses. Rather than initiating classical immunity, epithelial antigen presentation is increasingly recognized as a mechanism for fine-tuning CD4^+^ T-cell responses within the tissue microenvironment. Understanding how this unconventional antigen-presenting system is regulated, and how it contributes to intestinal homeostasis and disease, has therefore become a central question in mucosal immunology.

## 3. MHC-II Antigen Presentation Machinery in Intestinal Epithelial Cells

Antigen presentation through major histocompatibility complex class II (MHC-II) is traditionally regarded as a defining feature of professional antigen-presenting cells (APCs), including dendritic cells, macrophages, and B lymphocytes. These cells constitutively express the molecular machinery required to process exogenous proteins, load antigenic peptides onto MHC-II molecules, and activate antigen-specific CD4^+^ T cells [6,55,56]. In contrast, most non-hematopoietic cells either lack MHC-II expression or acquire it only under inflammatory conditions. The discovery that intestinal epithelial cells (IECs) express MHC-II therefore challenged the classical view of antigen presentation and raised fundamental questions regarding the purpose and functionality of this pathway at mucosal surfaces.

Importantly, expression of MHC-II alone does not confer antigen-presenting capacity. Productive antigen presentation requires a coordinated network of accessory molecules responsible for antigen uptake, intracellular processing, peptide loading, and transport of peptide–MHC complexes to the cell surface. Consequently, determining whether IECs possess the complete antigen-processing machinery has been central to understanding whether these cells function merely as MHC-II-expressing epithelial cells or as bona fide non-conventional APCs.

The canonical MHC-II pathway begins in the endoplasmic reticulum, where newly synthesized MHC-II α- and β-chains assemble with the invariant chain (CD74). Beyond stabilizing nascent MHC-II molecules, CD74 occupies the peptide-binding groove, preventing premature peptide loading within the endoplasmic reticulum and directing the complex toward the endosomal compartment. During endosomal maturation, progressive proteolytic degradation of CD74 generates the class II-associated invariant chain peptide (CLIP), which remains bound to the peptide-binding groove until it is exchanged for antigenic peptides through the catalytic activity of HLA-DM (H2-M in mice). These peptides are generated predominantly through proteolytic degradation of endocytosed proteins, although autophagy and other intracellular trafficking pathways can also contribute to the peptide repertoire. Following peptide loading, stable MHC-II–peptide complexes are transported to the plasma membrane, where they become available for recognition by [57,58].

Early studies investigating epithelial MHC-II largely focused on surface expression of HLA-DR molecules in human intestinal biopsies [59,60], leaving uncertain whether IECs possessed the intracellular machinery required for antigen processing. Subsequent work substantially expanded this view by demonstrating that epithelial cells express multiple components of the canonical MHC-II pathway, including CD74, HLA-DM, lysosomal proteases, and class II transactivator (CIITA), the master transcriptional regulator of MHC-II gene expression [12]. Human intestinal epithelial cell lines exposed to IFNγ acquire the capacity to internalize extracellular proteins, process intact antigens, and present antigenic peptides to CD4^+^ T cells [55,61], indicating that epithelial cells are capable of executing the complete MHC-II processing pathway rather than simply displaying preformed peptide–MHC complexes.

Recent studies using primary intestinal organoids have provided further support for this concept. Unlike transformed epithelial cell lines, organoids preserve the cellular identity and differentiation programs of primary IECs, allowing direct assessment of epithelial-intrinsic functions independent of contaminating professional APCs [62]. Human and murine intestinal organoids respond robustly to IFNγ stimulation by inducing coordinated expression of MHC-II genes together with antigen-processing components, confirming that the epithelial compartment itself contains the molecular machinery necessary for antigen presentation [8,14,55]. Moreover, these systems have established that epithelial MHC-II expression is not permanently fixed but rather represents a highly plastic program that depends upon continuous environmental stimulation.

Although the core antigen-processing pathway appears largely conserved between professional APCs and IECs, several important differences likely distinguish epithelial antigen presentation. Professional APCs continuously sample extracellular proteins through phagocytosis, macropinocytosis, and receptor-mediated endocytosis, subsequently migrating to secondary lymphoid organs where they initiate naïve T-cell priming [63]. In contrast, IECs are sessile cells positioned directly at the luminal interface, where they encounter dietary proteins, microbial products, and epithelial-derived material within a highly specialized microenvironment. Rather than initiating systemic immune responses, epithelial antigen presentation is thought to operate locally within the intestinal mucosa, where antigen-experienced lymphocytes predominate [55,62]. Consequently, the repertoire of peptides presented by IECs, the mechanisms through which luminal antigens access intracellular processing compartments, and the responding T-cell populations are likely to differ substantially from those associated with professional APCs.

The sources of antigens available for epithelial presentation remain incompletely understood. IECs can internalize soluble luminal proteins through endocytic pathways, while autophagy may provide endogenous peptides derived from cytosolic proteins or damaged organelles [58,64]. Continuous epithelial turnover further exposes intracellular proteins that may contribute to self-antigen presentation [55]. In addition, specialized epithelial populations, including M cells and goblet cells, participate in antigen transport across the epithelial barrier [65,66], raising the possibility that epithelial subsets differ not only in MHC-II expression but also in the spectrum of antigens they process and present. Defining the origin and composition of the epithelial immunopeptidome has therefore emerged as an important frontier in understanding epithelial antigen presentation.

Another distinguishing feature of IECs is their relatively limited expression of classical co-stimulatory molecules. Although inflammatory conditions can induce expression of CD80, CD86, CD40, and other accessory molecules, healthy epithelial cells generally express these proteins at low levels [62,67]. Conversely, IECs express several co-inhibitory and immunomodulatory molecules, including PD-L1 [55], suggesting that epithelial antigen presentation may be intrinsically biased toward modulation rather than initiation of adaptive immune responses [55,62,67]. These observations have fueled long-standing debate regarding whether epithelial MHC-II primarily promotes immune activation, reinforces peripheral tolerance, or dynamically performs both functions depending on the surrounding inflammatory context.

Collectively, current evidence supports the concept that intestinal epithelial cells possess the molecular machinery required for authentic MHC-II-mediated antigen presentation. Rather than representing incomplete or aberrant expression of isolated MHC-II molecules, epithelial antigen presentation appears to be a tightly regulated and environmentally responsive program. However, important questions remain unresolved, including the identity of physiologically relevant antigens, the epithelial subsets that contribute most prominently to antigen presentation, and the precise mechanisms through which epithelial MHC-II shapes CD4^+^ T-cell responses in vivo. Addressing these questions will require integration of emerging technologies, including single-cell transcriptomics, spatial biology, organoid systems, and immunopeptidomics, which together promise to redefine our understanding of antigen presentation at epithelial barriers.

## 4. Regional and Epithelial Subset Specificity of MHC-II Expression

**Figure 2:**
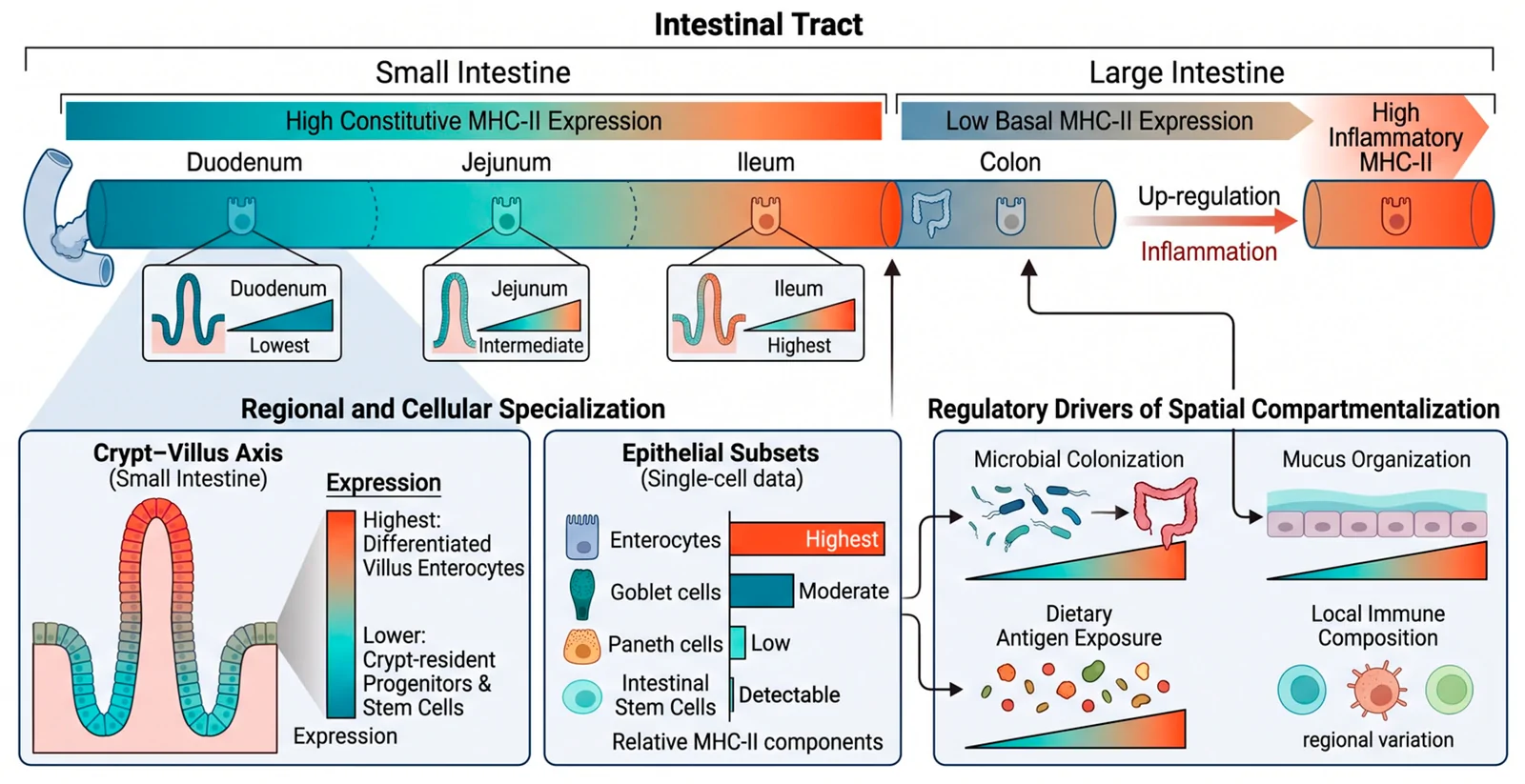
Spatial organization of epithelial MHC-II expression along the intestinal tract. Epithelial MHC-II expression exhibits regional and cellular specialization throughout the intestine. Under homeostatic conditions, constitutive MHC-II expression is most prominent in the small intestine, whereas the healthy colon displays comparatively low basal expression that becomes markedly upregulated during inflammation. Within the small intestine, MHC-II expression varies along the crypt–villus axis, with differentiated villus enterocytes exhibiting the highest expression while crypt-resident progenitors and stem cells display lower but detectable levels. Recent single-cell transcriptomic studies have revealed that multiple epithelial subsets—including enterocytes, goblet cells, Paneth cells, and intestinal stem cells—express components of the MHC-II antigen-processing machinery to varying degrees. Regional differences in microbial colonization, mucus organization, dietary antigen exposure, and local immune composition further contribute to the spatial regulation of epithelial antigen presentation. Together, these findings support a model in which epithelial MHC-II is compartmentalized according to anatomical location, epithelial lineage, and tissue-specific immune requirements.

One of the most significant advances in the study of epithelial MHC-II has been the recognition that antigen presentation is not uniformly distributed throughout the intestinal epithelium (Figure 2). Rather than representing a universal feature of all intestinal epithelial cells (IECs), MHC-II expression exhibits remarkable spatial heterogeneity across anatomical regions, epithelial lineages, and differentiation states [62]. This regional specialization is consistent with the diverse physiological functions performed along the gastrointestinal tract and suggests that epithelial antigen presentation has evolved to meet distinct immunological demands within different tissue microenvironments.

Under homeostatic conditions, constitutive epithelial MHC-II expression is most prominent in the small intestine [6,7], whereas expression in the healthy colon is comparatively limited [12]. This regional distribution likely reflects the fundamentally different ecological landscapes encountered by these tissues. The small intestine is continuously exposed to newly ingested dietary antigens, relatively low-density but highly immunogenic microbial communities, and an extensive network of Peyer’s patches and isolated lymphoid follicles that support adaptive immune responses [62,68]. In contrast, the colon contains a substantially larger microbial biomass but is protected by a thick mucus layer that physically separates luminal microorganisms from the epithelial surface [68]. These anatomical and functional differences create distinct patterns of epithelial–immune communication and likely explain why epithelial MHC-II expression is maintained constitutively in the small intestine but remains relatively restricted within the healthy colon.

Importantly, this regional organization is not static. During inflammatory conditions, including inflammatory bowel disease, enteric infection, and graft-versus-host disease, MHC-II expression expands dramatically throughout the intestinal tract, including colonic epithelial cells that normally exhibit little constitutive [7,62]. These observations indicate that regional differences primarily reflect differences in local regulatory signals rather than intrinsic limitations of individual epithelial populations. Indeed, colonic epithelial cells retain the capacity to robustly induce MHC-II following exposure to IFNγ and other inflammatory stimuli, highlighting the remarkable plasticity of the epithelial antigen-presentation program [12,69].

Spatial heterogeneity extends beyond differences between the small intestine and colon. Within the small intestine itself, epithelial MHC-II expression varies considerably along the crypt–villus axis [70]. Early histological studies demonstrated that differentiated enterocytes located near the villus tip exhibit the highest levels of surface MHC-II expression, whereas cells within the proliferative crypt compartment display lower expression under basal conditions [70,71]. Because epithelial differentiation is accompanied by profound transcriptional and metabolic remodeling, these observations initially suggested that MHC-II expression represented a feature of terminally differentiated absorptive enterocytes. However, recent genetic and single-cell approaches have challenged this simplified model [72].

High-resolution single-cell transcriptomic analyses have revealed that MHC-II expression is distributed across multiple epithelial lineages rather than being restricted to mature enterocytes [72]. In addition to absorptive epithelial cells, subsets of stem cells, transit-amplifying progenitors, secretory populations, and differentiated epithelial cells express components of the antigen-processing machinery, although with substantial quantitative variation [8]. These findings indicate that epithelial antigen presentation is regulated according to both lineage identity and differentiation state. Moreover, expression of CIITA, CD74, H2-Ab1 (or HLA-DRA in humans), and other components of the MHC-II pathway is not always coordinated to the same extent across epithelial subsets, suggesting that individual cell populations may differ in their capacity to process and present antigen.

Particularly intriguing is the observation that intestinal stem cells residing at the crypt base express MHC-II under physiological conditions. Initial studies identified constitutive MHC-II expression by Lgr5^+^ intestinal stem cells, raising the possibility that antigen presentation may influence stem-cell biology in addition to adaptive immunity [8]. Because stem cells continuously regenerate the intestinal epithelium, immune-mediated regulation of this compartment could provide an important mechanism through which microbial and inflammatory signals influence epithelial renewal and tissue repair. Although the precise function of stem-cell MHC-II remains incompletely understood, emerging evidence suggests that immune-derived cytokines and interactions with neighboring lymphocytes contribute to regulating stem-cell proliferation, differentiation, and regenerative capacity [8,73]. Whether antigen presentation itself directly participates in these processes remains an important question for future investigation.

The advent of single-cell RNA sequencing and spatial transcriptomics has transformed our understanding of epithelial MHC-II regulation. Earlier studies relied largely on bulk tissue analyses or immunohistochemistry, approaches that were unable to distinguish epithelial subsets or resolve regional differences within individual crypt–villus units. In contrast, single-cell atlases have revealed extensive epithelial diversity, identifying transcriptionally distinct epithelial populations with specialized immune functions distributed along both the proximal–distal and crypt–villus axes [8,72]. These datasets consistently demonstrate that expression of MHC-II genes is highly heterogeneous and dynamically regulated according to epithelial identity, anatomical location, and inflammatory status. Furthermore, spatial transcriptomic approaches now permit simultaneous visualization of epithelial MHC-II expression together with neighboring immune populations, offering unprecedented insight into the local cellular interactions that govern epithelial antigen presentation [6,74].

The increasing appreciation of epithelial heterogeneity has important implications for interpreting both experimental and clinical studies. Many earlier investigations quantified epithelial MHC-II using bulk tissue samples or total epithelial isolates, implicitly assuming that changes reflected uniform regulation across all IECs [62]. It is now evident that alterations in overall MHC-II expression may instead arise from selective expansion, contraction, or activation of specific epithelial subsets [72]. Likewise, disease-associated changes in epithelial antigen presentation may reflect regional remodeling of epithelial composition rather than simple transcriptional upregulation. Future studies integrating lineage tracing, organoid systems, single-cell multi-omics, and spatial biology will therefore be essential for defining the precise epithelial populations responsible for antigen presentation in health and disease.

Collectively, these findings support a revised view of epithelial MHC-II as a spatially organized immune program rather than a uniform epithelial characteristic. Regional differences between the small intestine and colon, compartmentalization along the crypt–villus axis, and lineage-specific transcriptional programs together generate a highly specialized landscape of epithelial antigen presentation. Understanding how this spatial organization intersects with microbial colonization, dietary signals, and immune-cell interactions will be critical for defining the physiological functions of epithelial MHC-II and for identifying the epithelial populations most relevant to intestinal homeostasis and inflammatory disease.

## 5. Regulation of Epithelial MHC-II

### 5.1. Transcriptional Control of Epithelial MHC-II Expression

**Figure 3:**
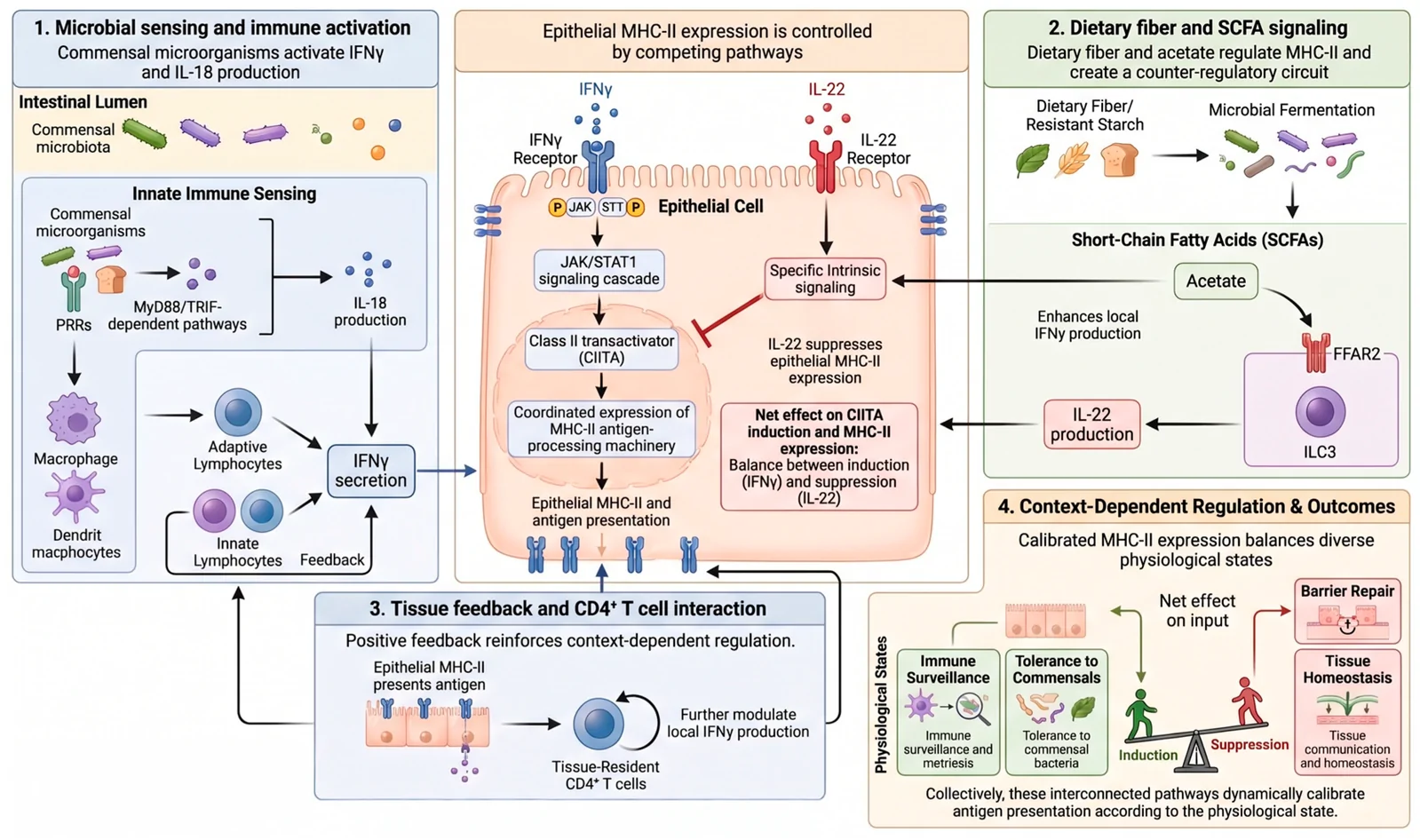
Integrated regulatory network controlling epithelial MHC-II expression. Epithelial MHC-II expression is dynamically regulated through the integration of microbial, dietary, immune, and epithelial-intrinsic signaling pathways. Commensal microorganisms activate innate immune sensing through pattern-recognition receptors (PRRs) and MyD88/TRIF-dependent pathways, promoting IL-18 production and IFNγ secretion by innate and adaptive lymphocytes. IFNγ activates the epithelial JAK/STAT1 signaling cascade, inducing the class II transactivator (CIITA) and coordinated expression of the MHC-II antigen-processing machinery. Dietary fiber further influences epithelial antigen presentation indirectly through microbial fermentation and the generation of short-chain fatty acids (SCFAs), particularly acetate. Acetate promotes epithelial MHC-II expression by enhancing local IFNγ production, while simultaneously activating FFAR2 signaling in ILC3s to stimulate IL-22 production. In contrast to IFNγ, IL-22 suppresses epithelial MHC-II expression, establishing a counter-regulatory circuit that limits excessive epithelial antigen presentation during tissue repair. Additional feedback interactions between epithelial MHC-II and tissue-resident CD4^+^ T cells further modulate local IFNγ production, reinforcing context-dependent regulation of antigen presentation. Collectively, these interconnected pathways enable intestinal epithelial cells to integrate dietary, microbial, and inflammatory cues, dynamically calibrating epithelial MHC-II expression to balance immune surveillance, tolerance, barrier repair, and tissue homeostasis.

The expression of major histocompatibility complex class II (MHC-II) by intestinal epithelial cells (IECs) is highly dynamic and tightly regulated at the transcriptional level [75]. Unlike professional antigen-presenting cells (APCs), which constitutively express MHC-II to support continuous immune surveillance, epithelial cells exhibit remarkable plasticity, rapidly adapting antigen-presentation programs in response to environmental and inflammatory cues [55]. Consequently, epithelial MHC-II should not be viewed as a fixed phenotypic marker, but rather as a context-dependent transcriptional program that integrates signals derived from the intestinal microenvironment (Figure 3).

At the center of this regulatory network is the class II transactivator (CIITA), widely recognized as the master regulator of MHC-II gene expression [76,77]. CIITA does not directly bind DNA; instead, it functions as a non-DNA-binding transcriptional coactivator that coordinates assembly of the enhanceosome on conserved regulatory elements within MHC-II promoters [77,78]. By recruiting chromatin-remodeling complexes and transcriptional machinery, CIITA drives coordinated expression of genes encoding MHC-II α- and β-chains together with multiple components of the antigen-processing pathway, including CD74 (invariant chain), HLA-DM (H2-M in mice), and accessory molecules required for peptide loading [76,77,78]. As a result, induction of CIITA establishes a complete antigen-presentation program rather than simply increasing surface expression of MHC-II.

CIITA expression itself is regulated through multiple promoter regions that confer cell type-specific responsiveness. In professional APCs, constitutive MHC-II expression is maintained primarily through promoter I and promoter III, whereas non-hematopoietic cells, including intestinal epithelial cells, rely predominantly on promoter IV (pIV) [79,80]. Activation of pIV is strongly dependent on interferon-γ (IFNγ), explaining why epithelial MHC-II expression is exquisitely sensitive to changes in the local inflammatory environment [69]. This distinction provides an important conceptual framework for understanding epithelial antigen presentation: whereas dendritic cells are intrinsically programmed to express MHC-II, epithelial cells acquire this capacity in response to tissue-derived signals [62,79].

Among the cytokines regulating epithelial MHC-II, IFNγ remains the dominant and best-characterized inducer [75]. Binding of IFNγ to its heterodimeric receptor activates the Janus kinase (JAK1/JAK2)-signal transducer and activator of transcription 1 (STAT1) signaling cascade, resulting in STAT1 phosphorylation, dimerization, and nuclear translocation [81,82]. Activated STAT1 induces interferon regulatory factor 1 (IRF1), which subsequently drives CIITA transcription through promoter IV [83,84]. The resulting increase in CIITA coordinates expression of the entire MHC-II antigen-processing machinery, enabling epithelial cells to process and present peptide antigens to CD4^+^ T cells [84,85]. Pharmacological inhibition or genetic disruption of components of the JAK/STAT pathway markedly reduces epithelial MHC-II expression, underscoring the central importance of this signaling axis [86,87].

Evidence from primary epithelial organoids has further established that activation of the IFNγ-JAK/STAT1-CIITA pathway is an epithelial-intrinsic response [75]. Human and murine intestinal organoids rapidly upregulate MHC-II genes following IFNγ stimulation in the absence of immune cells, demonstrating that epithelial cells possess all of the molecular components necessary to activate this transcriptional program autonomously [75,88]. Conversely, prolonged culture of intestinal organoids in the absence of inflammatory signals results in progressive loss of MHC-II expression, indicating that continuous environmental stimulation is required to sustain epithelial antigen presentation [89]. These observations emphasize that epithelial MHC-II reflects ongoing communication between the tissue microenvironment and epithelial cells rather than irreversible lineage commitment.

Although IFNγ is sufficient to induce epithelial MHC-II experimentally, accumulating evidence suggests that homeostatic expression within the intestine is more complex than a simple cytokine response. Under steady-state conditions, MHC-II is constitutively expressed by epithelial cells of the healthy small intestine despite the absence of overt inflammation [59,71,90,91]. Germ-free mice, antibiotic-treated animals, and mice deficient in microbial sensing pathways exhibit markedly reduced epithelial MHC-II expression, indicating that physiological antigen presentation depends upon continuous signals originating from the intestinal microbiota [9,73,88]. Rather than being produced during classical inflammation, low levels of IFNγ generated by resident immune populations—including intraepithelial lymphocytes, innate lymphoid cells, natural killer cells, and antigen-experienced T cells—appear sufficient to maintain basal CIITA activity in epithelial cells [11]. Thus, homeostatic epithelial MHC-II expression likely reflects tonic cytokine signaling within a healthy intestinal ecosystem rather than constitutive epithelial gene expression.

The distinction between homeostatic and inducible MHC-II expression has important biological implications. During homeostasis, epithelial MHC-II expression is largely restricted to the small intestine, where microbial density, nutrient absorption, and dietary antigen exposure create unique immunological demands [62,92]. In contrast, inflammatory conditions including inflammatory bowel disease (IBD), celiac disease, graft-versus-host disease (GVHD), and enteric infections are characterized by robust IFNγ production, resulting in widespread induction of epithelial MHC-II throughout both the small intestine and colon [93,94,95,96,97]. Accordingly, epithelial antigen presentation should not be considered a binary phenomenon but rather a continuum whose magnitude and anatomical distribution are dynamically shaped by the local cytokine milieu.

Species-specific differences further complicate interpretation of epithelial MHC-II biology. Healthy human small intestinal epithelial cells constitutively express abundant HLA-DR, with expression increasing toward the villus tip, whereas colonic epithelial expression is generally low under physiological conditions and increases markedly during inflammation [55,98]. Murine studies demonstrate a broadly similar regional pattern but have additionally identified constitutive MHC-II expression within intestinal stem cells and other crypt-resident epithelial populations, observations made possible by genetic reporter systems and single-cell transcriptomics [7,9]. Although the overall regulatory principles appear highly conserved, differences in epithelial turnover, microbial composition, and immune architecture between mice and humans likely influence both the magnitude and cellular distribution of epithelial MHC-II expression [9,62]. Careful consideration of these species-specific differences will therefore be essential when translating mechanistic findings from experimental models to human disease.

Together, current evidence supports a model in which epithelial MHC-II expression is governed by a highly plastic transcriptional network centered on CIITA and regulated predominantly through IFNγ-dependent activation of the JAK/STAT1 pathway. Rather than functioning as constitutive antigen-presenting cells, intestinal epithelial cells continuously adjust their antigen-presentation capacity according to the immunological context of the surrounding tissue. This dynamic regulation enables epithelial cells to integrate environmental, microbial, and inflammatory signals into a coordinated adaptive immune response, providing the mechanistic foundation for the diverse regulatory pathways discussed in the following sections.

### 5.2. Immune-Cell-Derived Regulation of Epithelial MHC-II

Although activation of the IFNγ–CIITA signaling axis provides the molecular framework for epithelial MHC-II expression [55,88], the upstream signals that initiate this program originate primarily from the surrounding immune compartment [99]. The intestinal epithelium exists in constant dialogue with resident leukocytes [3], and epithelial antigen presentation should therefore be viewed as an emergent property of epithelial–immune crosstalk rather than an isolated epithelial process [55]. Immune cells continuously sense microbial colonization, tissue damage, and inflammatory cues, translating these signals into cytokine networks that determine the magnitude, anatomical distribution, and duration of epithelial MHC-II expression [3,99]. Consequently, the regulation of epithelial antigen presentation reflects the collective activity of the intestinal immune ecosystem [3,88].

Among the cytokines implicated in this process, interferon-γ (IFNγ) remains the dominant regulator of epithelial MHC-II expression [12,55]. Numerous studies have demonstrated that IFNγ is both necessary and sufficient to induce coordinated expression of MHC-II molecules together with the antigen-processing machinery in intestinal epithelial cells [10,12]. Exposure of primary intestinal organoids or epithelial cell lines to IFNγ rapidly activates the JAK/STAT1-CIITA pathway, resulting in robust transcription of MHC-II genes [100,101]. Conversely, disruption of IFNγ signaling markedly impairs epithelial MHC-II expression, highlighting its central role as the principal cytokine linking immune activation to epithelial antigen presentation [12,86].

Importantly, the source of IFNγ differs substantially between homeostasis and inflammation [55]. During inflammatory conditions such as inflammatory bowel disease (IBD), graft-versus-host disease (GVHD), enteric infections, or celiac disease, activated Th1 cells and cytotoxic lymphocytes generate large quantities of IFNγ, driving widespread induction of epithelial MHC-II throughout the intestine [12,55,73,102]. In contrast, constitutive MHC-II expression observed in the healthy small intestine occurs in the absence of overt inflammation, suggesting that epithelial cells respond to relatively low concentrations of IFNγ generated under physiological conditions [103,104]. Rather than representing an inflammatory signal per se, tonic IFNγ production appears to function as a homeostatic regulator of epithelial immune competence [105,106].

Multiple immune populations have been proposed as sources of this homeostatic IFNγ. Intraepithelial lymphocytes (IELs), strategically positioned within the epithelial layer, represent an attractive candidate because of their intimate anatomical association with epithelial cells [107,108]. These lymphocytes continuously monitor epithelial integrity and rapidly respond to microbial or cellular stress, allowing localized delivery of IFNγ directly to neighboring IECs [108]. Similarly, innate lymphoid cells (ILCs), particularly type 1 innate lymphoid cells (ILC1s), reside within the intestinal mucosa and constitutively produce IFNγ in response to cytokines released by myeloid cells [106,109,110]. Natural killer (NK) cells and natural killer T (NKT) cells may further contribute to epithelial MHC-II regulation, particularly during early phases of infection when adaptive immune responses have not yet developed [89,111,112]. Together, these innate immune populations provide a rapid mechanism through which epithelial antigen presentation can be adjusted according to changes in the local tissue environment.

Adaptive T cells also play a central role in regulating epithelial MHC-II expression. Antigen-experienced CD4^+^ T cells constitute a major source of IFNγ within the intestinal mucosa and are increasingly recognized as important regulators of epithelial function beyond their classical effector activities [12,113,114]. Experimental models lacking adaptive lymphocytes exhibit markedly reduced epithelial MHC-II expression, indicating that continuous communication between epithelial cells and tissue-resident T cells contributes to maintaining antigen presentation under steady-state conditions [11,12,115]. During chronic inflammation, expansion of IFNγ-producing Th1 cells further amplifies epithelial MHC-II expression, increasing both the intensity and anatomical distribution of antigen presentation throughout the intestinal epithelium [7,116,117,118].

Recent work has expanded this regulatory framework by identifying interleukin-18 (IL-18) as an important upstream regulator of epithelial MHC-II expression [10]. IL-18, a member of the IL-1 cytokine family processed through inflammasome activation, is constitutively produced within the intestine and serves as a potent inducer of IFNγ production by multiple lymphocyte populations [119]. Elegant genetic studies demonstrated that microbiota-induced IL-18 promotes epithelial MHC-II indirectly by stimulating IFNγ production rather than acting directly on epithelial cells [10,120,121]. This finding significantly advanced the field by linking microbial sensing, inflammasome activation, innate lymphocytes, and epithelial antigen presentation into a single regulatory pathway. Rather than functioning independently, IL-18 and IFNγ therefore operate within a hierarchical cytokine cascade that couples microbial colonization to epithelial immune adaptation [89,122].

One of the emerging concepts in epithelial MHC-II biology is that its regulation is reinforced through positive feedback circuits between epithelial and immune cells. Initial microbial or inflammatory signals activate resident immune populations to produce IFNγ, inducing epithelial MHC-II expression through CIITA [12]. Once expressed, epithelial MHC-II can directly modulate antigen-experienced CD4^+^ T cells and intraepithelial lymphocytes residing within the mucosa [9,11]. Activated lymphocytes subsequently produce additional IFNγ, reinforcing epithelial MHC-II expression and sustaining local antigen presentation [7]. Similar amplification loops may involve IL-18, which promotes IFNγ secretion by innate and adaptive lymphocytes, thereby further enhancing epithelial MHC-II expression [12]. These reciprocal interactions suggest that epithelial antigen presentation is maintained through dynamic feed-forward circuits rather than transient cytokine exposure alone.

Such feedback mechanisms are likely advantageous during acute infection, allowing rapid amplification of local immune responses while restricting systemic inflammation [7,12,123]. However, persistent activation of these same circuits may become maladaptive during chronic inflammatory disorders [62,116]. Sustained IFNγ production can maintain high levels of epithelial MHC-II expression, potentially perpetuating interactions between epithelial cells and pathogenic T-cell populations [86,115]. Conversely, interruption of these feedback loops may contribute to restoration of epithelial homeostasis following resolution of inflammation [14,124]. Understanding how these regulatory circuits are initiated, amplified, and ultimately terminated remains an important challenge for the field.

In contrast to IFNγ, which potently induces epithelial MHC-II expression, IL-22 functions as a counter-regulatory cytokine that restrains epithelial antigen presentation. Produced primarily by ILC3s and, under certain conditions, Th17 cells, IL-22 is well established as a central regulator of epithelial proliferation, antimicrobial peptide production, mucus secretion, and tissue repair [125,126]. Recent studies have demonstrated that IL-22 also directly suppresses IFNγ-induced MHC-II expression in epithelial cells by downregulating both CIITA, the master regulator of MHC-II transcription, and H2-Aα, while partially limiting endoplasmic reticulum (ER) stress, a process linked to epithelial MHC-II induction [127]. Consistent with these findings, mice lacking the epithelial IL-22 receptor (IL-22RA1) display constitutively elevated epithelial MHC-II expression, supporting a physiological role for IL-22 in limiting epithelial antigen presentation under homeostatic conditions [127]. Interestingly, the antagonistic relationship between IL-22 and IFNγ appears to extend beyond the intestine. In human bronchial epithelial cells, IL-22 suppresses IFNγ-induced expression of MHC-I, MHC-II, ICAM-1, and multiple pro-inflammatory chemokines while simultaneously promoting epithelial migration and wound repair [128]. These observations suggest that IL-22 does not simply inhibit individual inflammatory genes but instead promotes a broader epithelial repair program that counterbalances IFNγ-driven immune activation across mucosal barrier tissues. Within the intestine, this regulatory axis likely enables epithelial cells to transition from an inflammatory state characterized by enhanced antigen presentation toward a regenerative program that restores barrier integrity following tissue injury.

The opposing effects of IFNγ and IL-22 highlight the remarkable plasticity of epithelial antigen presentation. Rather than being governed by a single cytokine, epithelial MHC-II expression reflects the integration of competing inflammatory and tissue-reparative signals within the local microenvironment. This balance may be further influenced by environmental cues. For example, microbiota-derived acetate has recently been shown to simultaneously enhance local IFNγ production [129,130,131] while activating FFAR2 signaling in ILC3s to promote IL-22 secretion [14,132,133]. These seemingly opposing pathways converge on the intestinal epithelium, where IFNγ promotes CIITA-dependent MHC-II expression whereas IL-22 restrains the same transcriptional program, establishing a dynamic regulatory circuit that fine-tunes epithelial antigen presentation according to the physiological state of the tissue.

Collectively, current evidence supports a model in which epithelial MHC-II expression is orchestrated by immune-cell-derived cytokines rather than regulated exclusively within epithelial cells. IFNγ functions as the principal inducer of epithelial antigen presentation by activating the JAK/STAT1–CIITA transcriptional program, whereas IL-18 amplifies this pathway indirectly through stimulation of IFNγ-producing innate and adaptive lymphocytes. In contrast, IL-22 acts as a counter-regulatory cytokine that restrains epithelial MHC-II expression by suppressing CIITA and MHC-II gene expression while promoting epithelial repair and barrier restoration. The integration of these opposing cytokine signals establishes a dynamic regulatory network that continuously calibrates epithelial antigen presentation according to the physiological state of the intestinal mucosa. Bidirectional communication between epithelial cells and immune populations further reinforces this regulation through feedback circuits that balance immune surveillance with tissue homeostasis. As discussed in the following sections, these immune-dependent pathways are themselves shaped by environmental cues—including the intestinal microbiota, dietary components, and microbial metabolites—which together determine the magnitude, spatial distribution, and functional consequences of epithelial MHC-II expression.

### 5.3. Microbiota-Dependent Regulation of Epithelial MHC-II

Among the diverse environmental factors regulating epithelial MHC-II expression, the intestinal microbiota has emerged as the dominant physiological determinant. While inflammatory cytokines such as IFNγ provide the immediate transcriptional stimulus for CIITA activation, accumulating evidence demonstrates that basal epithelial MHC-II expression depends on continuous microbial colonization [6,12]. Rather than representing an epithelial program that is constitutively active in healthy tissues, MHC-II expression is dynamically maintained through ongoing interactions between commensal microorganisms, the mucosal immune system, and the intestinal epithelium [9,88]. This concept has fundamentally changed our understanding of epithelial antigen presentation, establishing the microbiota not simply as a source of antigens but as a key regulator of the epithelial antigen presentation itself [134].

The importance of microbial colonization first became apparent through studies in germ-free mice. Compared with conventionally housed animals, germ-free mice exhibit a profound reduction in epithelial MHC-II expression throughout the small intestine despite the absence of overt epithelial abnormalities [7,9,135]. Colonization of germ-free animals with complex microbial communities restores epithelial MHC-II, demonstrating that microbial exposure is both necessary and sufficient to establish physiological antigen presentation [135,136]. These observations indicate that constitutive epithelial MHC-II expression is not developmentally programmed but instead requires continuous microbial stimulation throughout adult life [88]. Importantly, restoration of MHC-II following microbial colonization occurs rapidly, highlighting the remarkable plasticity of the epithelial antigen-presentation program [88,135]. Complementary evidence has been obtained through antibiotic-mediated depletion of the intestinal microbiota. Broad-spectrum antibiotic treatment markedly reduces epithelial MHC-II expression in adult mice, even after normal epithelial development has been established [7,73,137]. Unlike germ-free models, which cannot distinguish developmental from homeostatic effects, antibiotic studies demonstrate that the mature intestinal epithelium requires persistent microbial signals to maintain MHC-II expression [9,88]. Together, these findings establish that epithelial antigen presentation is continuously calibrated by the composition and abundance of the resident microbiota rather than being irreversibly determined during development.

How microbial colonization is translated into epithelial MHC-II expression has been the focus of intense investigation. Current evidence suggests that this regulation is largely indirect [9,88,138]. Rather than acting directly on epithelial cells, commensal microorganisms activate innate immune pathways that subsequently induce cytokine networks capable of stimulating epithelial antigen presentation [62,139,140]. Pattern-recognition receptors expressed by myeloid cells and epithelial cells recognize conserved microbial-associated molecular patterns, initiating signaling cascades that depend predominantly on the adaptor proteins myeloid differentiation primary response protein 88 (MyD88) and Toll/IL-1 receptor-domain-containing adaptor-inducing interferon-β (TRIF) [36,141]. Mice lacking components of these signaling pathways exhibit impaired epithelial MHC-II expression, indicating that continuous innate sensing of the microbiota is required to sustain homeostatic antigen presentation [7,88,142].

Importantly, not all members of the intestinal microbiota exert equivalent effects on epithelial MHC-II expression. Increasing evidence indicates that specific bacterial taxa possess a disproportionate capacity to modulate epithelial antigen presentation [9]. Among these, segmented filamentous bacteria (SFB) have emerged as one of the best-characterized examples. SFB intimately adhere to epithelial cells within the terminal ileum and are potent inducers of mucosal immune maturation, promoting differentiation of Th17 cells, induction of intraepithelial lymphocytes, and production of multiple cytokines involved in barrier immunity [143,144]. Recent studies demonstrated that SFB colonization markedly enhances epithelial MHC-II expression while simultaneously expanding cognate CD4^+^ intraepithelial lymphocytes [11]. Genetic disruption of epithelial MHC-II in this context altered bacteria-specific T-cell responses and impaired epithelial turnover, providing direct evidence that commensal-induced epithelial antigen presentation contributes to physiological host–microbiota interactions rather than representing a nonspecific consequence of microbial colonization [9,11].

These findings suggest that epithelial MHC-II is regulated not by microbial biomass alone but by the composition and immunological properties of individual microbial communities. Different bacterial species vary substantially in their capacity to activate innate immune pathways, induce cytokine production, adhere to the epithelial surface, or generate immunomodulatory metabolites [145,146]. Consequently, the intestinal microbiota should be viewed not as a single regulatory entity but as a diverse ecosystem whose individual members differentially shape epithelial immune function. This concept may help explain the considerable inter-individual variability in epithelial MHC-II expression observed among healthy individuals and may contribute to disease susceptibility associated with microbial dysbiosis.

Recent work further suggests that epithelial MHC-II participates in reciprocal communication between the host and its microbiota. Rather than functioning solely as a downstream target of microbial regulation, epithelial antigen presentation can itself influence microbial ecology by shaping bacteria-reactive CD4^+^ T-cell responses [9,11]. Conditional deletion of epithelial MHC-II alters the composition and activation state of mucosal T-cell populations, potentially influencing epithelial barrier integrity, antimicrobial defense, and ultimately the ecological landscape experienced by commensal microorganisms [9]. Thus, epithelial MHC-II may participate in a bidirectional feedback loop in which the microbiota regulates epithelial antigen presentation while epithelial antigen presentation simultaneously contributes to maintaining microbial homeostasis.

Despite these advances, several important questions remain unresolved. The microbial molecules responsible for initiating epithelial MHC-II induction have not been fully defined, nor is it clear whether bacterial adherence, metabolite production, or antigenic complexity represent the dominant stimulus. Similarly, although SFB has emerged as an informative experimental model, its limited abundance in adult humans raises important questions regarding the identity of human commensal species that perform analogous functions. Defining these microbial regulators, together with the immune pathways through which they influence epithelial antigen presentation, represents an important priority for future investigation.

Most mechanistic insights regarding microbiota-dependent regulation of epithelial MHC-II have been derived from mouse models, particularly studies involving segmented filamentous bacteria (SFB), a commensal organism that potently shapes intestinal immune responses in rodents. While these studies have established a central role for the microbiota in regulating epithelial antigen presentation, the translational relevance of SFB-driven mechanisms remains uncertain because SFB-like organisms are uncommon in adult human intestine. In humans, epithelial MHC-II expression is likely influenced by a more complex microbial ecosystem involving multiple bacterial taxa, microbial products, and host-derived cytokine networks. However, the specific microbial species and mechanisms responsible for regulating epithelial MHC-II in humans remain poorly defined and represent an important area for future investigation.

Collectively, these studies demonstrate that intestinal microbiota is a major regulator of epithelial MHC-II expression. Although SFB has provided an important experimental model for defining microbiota-dependent regulation in mice, whether analogous mechanisms operate in humans remains unclear.

### 5.4. Dietary Regulation of Epithelial MHC-II Through Microbial Metabolites

The intestinal microbiota does not function independently of its nutritional environment. Rather, microbial communities continuously adapt their composition and metabolic activity in response to dietary substrates, generating a diverse array of bioactive metabolites that profoundly influence epithelial physiology and mucosal immunity [147]. Consequently, dietary regulation of epithelial MHC-II should not be viewed as a pathway distinct from microbial regulation, but rather as an additional layer through which nutrient availability shapes host–microbiota interaction [148]. This concept has gained increasing attention in recent years, revealing that dietary components influence not only the composition of the microbiota but also its immunomodulatory capacity [149].

Among dietary factors, fermentable fiber has emerged as a major regulator of intestinal immune homeostasis. Diets enriched in complex carbohydrates promote the expansion of fiber-fermenting bacterial communities, which convert otherwise indigestible polysaccharides into short-chain fatty acids (SCFAs), including acetate, propionate, and butyrate [150]. Beyond serving as metabolic substrates for intestinal epithelial cells, SCFAs function as signaling molecules that regulate epithelial metabolism, barrier integrity, cytokine production, and immune-cell differentiation through both receptor-dependent and epigenetic mechanisms [151]. Numerous studies have demonstrated that SCFAs enhance epithelial barrier function by promoting tight junction assembly, stimulating epithelial renewal, increasing mucus production, and limiting excessive inflammatory responses through both epithelial- and immune-cell-intrinsic pathways [152]. Collectively, these observations establish microbial metabolites as key mediators linking dietary composition to mucosal immune homeostasis.

Until recently, however, whether microbial metabolites directly influenced epithelial antigen presentation remained largely unexplored. Most studies investigating epithelial MHC-II focused on inflammatory cytokines or microbial colonization, implicitly assuming that dietary effects occurred indirectly through changes in microbial composition. Emerging evidence has challenged this view by demonstrating that microbial metabolism itself represents an important determinant of epithelial MHC-II expression.

Interestingly, acetate has emerged as a key regulator of epithelial MHC-II expression, acting in a region-specific manner. In the small intestine, acetate promotes epithelial MHC-II expression through an IFNγ-dependent pathway, supporting epithelial antigen presentation under homeostatic conditions [6]. In contrast, acetate suppresses epithelial MHC-II expression in the colon by stimulating FFAR2-dependent IL-22 production by ILC3s, thereby favoring epithelial repair while limiting excessive antigen presentation during tissue regeneration [14]. These findings reveal that the same microbial metabolite can exert distinct—even opposing—effects depending on the local epithelial and immune microenvironment. They further underscore that epithelial MHC-II regulation is not a uniform process throughout the intestine but instead reflects the unique anatomical, microbial, and immunological characteristics of individual intestinal regions.

Importantly, acetate is unlikely to be the only microbial metabolite influencing epithelial MHC-II expression. Other SCFAs, including butyrate and propionate, possess well-established immunomodulatory properties and regulate epithelial physiology through G protein-coupled receptors, histone deacetylase inhibition, and metabolic reprogramming [150]. Likewise, secondary bile acids, indole derivatives generated through bacterial tryptophan metabolism, polyamines, and numerous other microbiota-derived metabolites have all been implicated in epithelial differentiation, barrier function, and immune regulation [146,153]. Whether these metabolites directly regulate epithelial antigen presentation, cooperate with acetate, or preferentially influence specific epithelial subsets remains unknown. Defining the relative contribution of individual microbial metabolites therefore represents an important area for future investigation.

These observations also illustrate the remarkable integration of nutritional, microbial, and immune signals within the intestinal ecosystem. Dietary fiber provides the substrate for microbial fermentation, the microbiota generates SCFAs and other bioactive metabolites, and these metabolites modulate local cytokine production by immune cells [154]. The resulting balance between IFNγ-driven induction and IL-22-mediated suppression ultimately determines the magnitude of epithelial MHC-II expression in a tissue-specific manner. Rather than functioning as independent regulatory pathways, diet, the microbiota, and the immune system therefore operate as interconnected components of a multilayered signaling network that continuously calibrates epithelial antigen presentation according to the physiological state of the intestinal mucosa.

The interaction between diet and epithelial MHC-II also has important implications for intestinal disease. Western dietary patterns characterized by reduced fiber intake and altered microbial metabolism have been associated with impaired epithelial barrier function, microbial dysbiosis, and increased susceptibility to chronic inflammatory disorders [155]. Recent study further demonstrates that Western diets are accompanied by reduced epithelial MHC-II expression and altered mucosal immune homeostasis [73]. Although the mechanisms underlying these observations remain incompletely understood, they likely reflect the combined effects of altered microbial composition, changes in microbial metabolite production, and disruption of immune-cell-derived cytokine networks. These findings suggest that dietary interventions capable of restoring beneficial microbial metabolism may provide a complementary strategy to modulate epithelial antigen presentation and improve mucosal immune function.

Collectively, current evidence supports a model in which dietary regulation of epithelial MHC-II is mediated primarily through microbial metabolism rather than direct nutrient sensing by epithelial cells. Microbial metabolites function as intermediaries that translate dietary information into immune-cell-derived cytokine responses, which ultimately regulate epithelial antigen presentation in a region-specific manner. More broadly, these findings establish epithelial MHC-II as an environmentally responsive immune program that integrates dietary composition, microbial metabolism, and local immune signals to continuously adapt epithelial immune function to the changing luminal environment. Understanding how additional microbial metabolites participate in this regulatory network will likely reveal new mechanisms through which nutritional status shapes adaptive immunity at epithelial barriers.

## 6. Functional Consequences of Epithelial MHC-II

The identification of MHC-II expression by intestinal epithelial cells (IECs) naturally raised a fundamental question: what is the physiological purpose of epithelial antigen presentation? Unlike professional antigen-presenting cells (APCs), IECs are strategically positioned at the interface between luminal antigens and the mucosal immune system but generally lack the migratory behavior and co-stimulatory capacity [17,67]. Consequently, the biological role of epithelial MHC-II has remained controversial for decades. Early studies alternately proposed that epithelial cells activate effector T cells, induce peripheral tolerance, or simply represent passive targets of inflammatory cytokines. Recent advances using conditional genetic models, organoid systems, and high-dimensional immune profiling have substantially refined this view. Rather than initiating adaptive immunity, epithelial MHC-II is increasingly recognized as a mechanism that fine-tunes local CD4^+^ T-cell responses, integrating environmental signals with tissue-specific immune [12,67,156].

One of the best-supported functions of epithelial MHC-II is the regulation of host–microbiota interactions. The intestinal immune system must maintain tolerance toward trillions of commensal microorganisms while remaining capable of mounting rapid responses against invasive pathogens. Because intestinal epithelial cells constitute the first cellular layer encountered by luminal microorganisms, epithelial antigen presentation is ideally positioned to continuously sample and communicate information regarding the microbial environment to neighboring lymphocytes [55]. Recent studies have demonstrated that epithelial MHC-II directly shapes bacteria-reactive CD4^+^ T-cell populations within the intestinal mucosa, thereby limiting excessive immune activation while preserving protective immunity. Conditional deletion of epithelial MHC-II results in altered activation of microbiota-specific T cells, supporting the concept that epithelial antigen presentation actively participates in maintaining immunological homeostasis rather than serving as a passive consequence of microbial colonization [6,156].

An important advance in recent years has been the identification of epithelial MHC-II as a regulator of intraepithelial CD4^+^ lymphocytes. Although intraepithelial lymphocytes (IELs) have traditionally been associated with cytotoxic CD8^+^ T cells [43], accumulating evidence indicates that specialized populations of CD4^+^ IELs also reside within the epithelial compartment and contribute to barrier integrity [157]. Elegant studies demonstrated that segmented filamentous bacteria (SFB)-induced epithelial MHC-II promotes the differentiation and maintenance of bacteria-specific CD4^+^ IELs, thereby coupling microbial colonization to epithelial immune adaptation. Loss of epithelial MHC-II disrupted these lymphocyte populations and impaired epithelial turnover, suggesting that antigen presentation contributes not only to immune regulation but also to epithelial tissue maintenance [6]. These findings highlight an emerging paradigm in which epithelial cells directly instruct resident lymphocyte populations responsible for preserving barrier homeostasis.

Beyond intraepithelial lymphocytes, epithelial MHC-II also regulates CD4^+^ T-cell responses within the lamina propria. Recent work demonstrated that antigen presentation by intestinal epithelial cells modulates bacteria-reactive conventional CD4^+^ T cells independently of professional APCs. Rather than inducing robust effector activation, epithelial antigen presentation appears to calibrate the magnitude and quality of local T-cell responses, limiting excessive inflammation while preserving antigen-specific immunity [6]. This regulatory function is particularly well suited to the intestinal environment, where immune cells continuously encounter abundant microbial and dietary antigens that require discrimination between harmless and pathogenic stimuli. Thus, epithelial MHC-II appears to function less as an initiator of immunity than as a local checkpoint that adjusts ongoing adaptive immune responses according to tissue context [55].

The mechanisms through which epithelial antigen presentation influences T-cell behavior remain incompletely understood. Unlike dendritic cells, IECs generally express relatively low levels of classical co-stimulatory molecules, suggesting that the outcome of antigen presentation depends heavily on the surrounding cytokine milieu and the activation state of responding lymphocytes [55,67]. Under homeostatic conditions, presentation of luminal antigens by epithelial cells may favor immune tolerance through maintenance of regulatory or hyporesponsive T-cell states [6]. In contrast, during inflammation, increased expression of MHC-II together with inflammatory cytokines may reinforce effector T-cell activation and antimicrobial immunity [12]. These observations support the emerging concept that epithelial MHC-II is intrinsically context-dependent, with its biological function determined by the surrounding tissue environment rather than by antigen presentation alone.

Another emerging function of epithelial MHC-II is the regulation of epithelial homeostasis itself. Although initially considered an exclusive immunological pathway, recent evidence indicates that epithelial antigen presentation influences epithelial turnover, differentiation, and regenerative capacity. Mice lacking epithelial MHC-II exhibit altered epithelial renewal following microbial colonization, suggesting that antigen presentation contributes to maintaining tissue integrity independently of its effects on adaptive immunity [6]. Furthermore, MHC-II expression by intestinal stem cells raises the intriguing possibility that immune-derived signals may directly influence stem-cell function through antigen-dependent interactions with neighboring lymphocytes [8]. Although the underlying mechanisms remain largely unknown, these findings suggest that epithelial antigen presentation participates in coordinating immune surveillance with epithelial regeneration.

The functional importance of epithelial MHC-II becomes particularly evident during enteric infection. Infection dramatically increases epithelial MHC-II expression through cytokine-dependent mechanisms, enhancing communication between epithelial cells and mucosal lymphocytes [12]. Experimental evidence indicates that epithelial antigen presentation contributes to protective immunity against intestinal pathogens by promoting localized CD4^+^ T-cell responses while preserving epithelial barrier integrity [6]. Recent work further demonstrated that dietary regulation of epithelial MHC-II through microbiota-derived acetate enhances resistance to *Clostridioides difficile* infection, linking nutritional status, microbial metabolism, and epithelial antigen presentation within a unified protective pathway [14]. Importantly, these findings suggest that epithelial MHC-II does not function solely during chronic inflammation but also represents a critical component of physiological host defense against enteric pathogens.

Despite these advances, the precise role of epithelial MHC-II remains incompletely resolved and may differ substantially according to anatomical location and disease context. During acute infection, enhanced epithelial antigen presentation likely amplifies protective immune responses while promoting tissue repair [6]. Conversely, persistent IFNγ production during chronic inflammatory disorders may maintain elevated epithelial MHC-II expression, potentially sustaining pathogenic interactions with activated T cells [12,156]. This association is particularly relevant in inflammatory bowel diseases such as ulcerative colitis and Crohn’s disease, where epithelial MHC-II expression is markedly increased in inflamed intestinal tissues and correlates with local immune activation [12,156]. Although this upregulation is generally viewed as a consequence of the pro-inflammatory cytokine milieu, persistent antigen presentation by intestinal epithelial cells may also influence disease progression by modulating mucosal CD4^+^ T-cell responses. However, whether epithelial MHC-II functions primarily as a driver of pathology or as a compensatory mechanism that attempts to restore immune homeostasis remains unclear. Rather than reflecting opposing biological functions, these seemingly contradictory observations likely illustrate the remarkable adaptability of epithelial antigen presentation. Similar to many pathways governing mucosal immunity, epithelial MHC-II appears to operate within a narrow physiological window in which both insufficient and excessive activity may compromise intestinal homeostasis.

**Figure 4:**
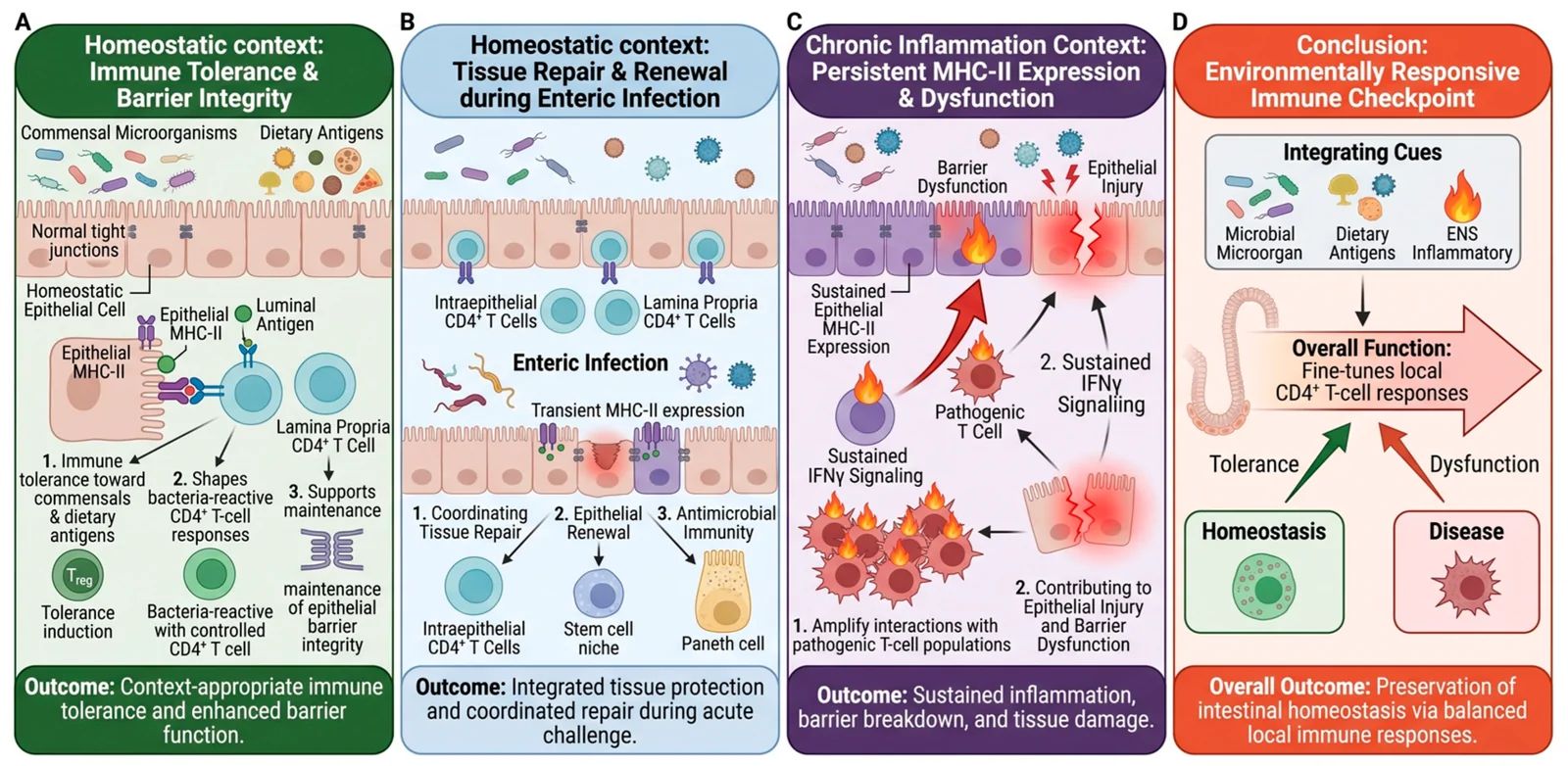
Functional consequences of epithelial MHC-II expression in intestinal homeostasis and disease. Epithelial MHC-II functions as a local regulator of adaptive immunity at the intestinal barrier. (**A**) Under homeostatic conditions, presentation of luminal antigens contributes to immune tolerance toward commensal microorganisms and dietary antigens, shapes bacteria-reactive CD4^+^ T-cell responses, and supports maintenance of epithelial barrier integrity. (**B**) During enteric infection, epithelial antigen presentation also promotes communication with intraepithelial and lamina propria CD4^+^ T cells, coordinating tissue repair, epithelial renewal, and antimicrobial immunity. (**C**) During chronic inflammation, sustained IFNγ signaling and persistent epithelial MHC-II expression may amplify interactions with pathogenic T-cell populations, contributing to epithelial injury and barrier dysfunction. Thus, the biological consequences of epithelial antigen presentation are highly context-dependent and are determined by the nature of environmental signals, the inflammatory milieu, and the responding immune cell populations. (**D**) Overall, epithelial MHC-II acts as an environmentally responsive immune checkpoint that integrates microbial, dietary, and inflammatory cues to fine-tune local CD4^+^ T-cell responses and preserve intestinal homeostasis.

Collectively, available evidence supports a revised view of epithelial MHC-II as a local regulator of adaptive immunity rather than a substitute for professional antigen-presenting cells. As summarized in Figure 4, epithelial MHC-II exerts context-dependent effects on intestinal immunity, promoting immune tolerance and barrier maintenance under homeostatic conditions while contributing to antimicrobial defense during infection and potentially amplifying pathogenic T-cell interactions during chronic inflammation. By continuously integrating microbial colonization, dietary signals, inflammatory cytokines, and epithelial differentiation programs, intestinal epithelial cells fine-tune CD4^+^ T-cell responses at the mucosal interface [6]. This regulatory capacity enables epithelial MHC-II to coordinate immune tolerance [156], antimicrobial defense [14], tissue repair [6], and barrier maintenance [8] according to the changing demands of the intestinal microenvironment. Understanding how these diverse functions become dysregulated in chronic intestinal disease is therefore critical for defining the therapeutic potential of targeting epithelial antigen presentation.

## 7. Epithelial MHC-II in Intestinal Disease

The growing appreciation that intestinal epithelial cells actively regulate local CD4^+^ T-cell responses has substantially altered our understanding of epithelial dysfunction in intestinal disease. Traditionally, epithelial MHC-II expression observed during inflammation was regarded as a secondary consequence of interferon-γ (IFNγ) exposure, serving primarily as a histological marker of immune activation. However, accumulating experimental evidence indicates that epithelial antigen presentation is not merely a bystander phenomenon but can actively influence disease progression by shaping tissue-specific immune responses. Importantly, the biological consequences of epithelial MHC-II appear to depend on the nature of the inflammatory stimulus, the responding immune populations, and the regional organization of the intestinal mucosa. Thus, epithelial MHC-II should be viewed as a context-dependent regulator of disease rather than as an inherently protective or pathogenic pathway.

### 7.1. Inflammatory Bowel Disease

Among chronic inflammatory disorders of the intestine, inflammatory bowel disease (IBD) has been most consistently associated with altered epithelial MHC-II expression. Increased HLA-DR expression by intestinal epithelial cells was first described several decades ago in patients with ulcerative colitis and Crohn’s disease [60] and has since become a well-recognized feature of active intestinal inflammation. Although this observation was initially interpreted as a consequence of elevated IFNγ within inflamed tissues, recent studies suggest that epithelial antigen presentation may also contribute to the perpetuation of mucosal immune responses.

IBD is characterized by profound disruption of epithelial barrier integrity, microbial dysbiosis, and excessive activation of mucosal CD4^+^ T cells [158]. Within this inflammatory environment, epithelial MHC-II expression expands beyond its normal regional distribution, becoming highly expressed throughout inflamed epithelial compartments [12]. Whether this response is beneficial or detrimental likely depends on disease stage. During the early phases of inflammation, increased epithelial antigen presentation may enhance communication with resident T cells and facilitate restoration of barrier homeostasis. However, sustained IFNγ production and chronic epithelial MHC-II expression may reinforce interactions with pathogenic effector T cells, contributing to persistent inflammation. These observations support the concept that epithelial MHC-II functions within a dynamic regulatory circuit whose outcome depends on the balance between tissue repair and immune-mediated injury.

### 7.2. Celiac Disease

Celiac disease provides another important example of dysregulated epithelial antigen presentation. Gluten-specific CD4^+^ T cells are central drivers of disease pathogenesis, and intestinal epithelial cells exhibit marked upregulation of HLA class II molecules within affected tissues [159]. Unlike IBD, where multiple microbial and immune pathways converge, celiac disease is characterized by a well-defined dietary antigen that elicits highly specific adaptive immune responses [13]. Enhanced epithelial MHC-II expression within the small intestine may therefore increase opportunities for local presentation of gluten-derived peptides and amplify interactions with antigen-experienced CD4^+^ T cells residing at the epithelial interface.

At the same time, celiac disease illustrates the complexity of interpreting epithelial MHC-II expression. Increased antigen presentation occurs in parallel with elevated IL-15 [160], IFNγ, epithelial stress responses, and expansion of cytotoxic intraepithelial lymphocytes [161], making it difficult to distinguish cause from consequence. Whether epithelial antigen presentation actively initiates pathogenic immunity or primarily reinforces ongoing inflammation remains incompletely resolved. Nevertheless, celiac disease highlights how disruption of normal epithelial–immune communication may contribute to chronic tissue injury when immune tolerance to dietary antigens is lost.

### 7.3. Graft-Versus-Host Disease

Compelling evidence supporting a direct pathogenic role for epithelial MHC-II has emerged from studies of gastrointestinal graft-versus-host disease (GVHD). Unlike many inflammatory disorders in which epithelial MHC-II expression correlates with disease activity, conditional genetic models have demonstrated that selective deletion of MHC-II from intestinal epithelial cells significantly attenuates GVHD severity following allogeneic hematopoietic stem cell transplantation [7]. These findings established that epithelial antigen presentation is not merely a marker of inflammation but can directly contribute to disease pathogenesis.

Mechanistically, donor-derived alloreactive CD4^+^ T cells recognize host MHC-II molecules expressed by intestinal epithelial cells, initiating localized immune activation and epithelial injury. Importantly, epithelial antigen presentation appears particularly relevant during the earliest phases of disease, when direct interactions between epithelial cells and infiltrating donor T cells amplify mucosal inflammation before widespread tissue destruction has occurred [7,162]. These studies provide some of the strongest experimental evidence that epithelial MHC-II can function as an active participant in disease rather than a passive target of inflammatory cytokines.

### 7.4. Enteric Infection

The role of epithelial MHC-II during enteric infection differs fundamentally from its role in chronic inflammatory disorders. Acute infection induces rapid upregulation of epithelial antigen presentation through cytokine-dependent mechanisms, enhancing communication between epithelial cells and mucosal lymphocytes at a time when rapid local immune responses are essential for pathogen control [12,62]. Rather than promoting chronic inflammation, transient induction of epithelial MHC-II appears to strengthen barrier immunity while coordinating tissue repair following infection-induced epithelial injury.

Recent studies have further demonstrated that epithelial MHC-II contributes to resistance against enteric pathogens by integrating environmental signals with adaptive immune regulation. Dietary fiber deficiency, microbial dysbiosis, or impaired microbial metabolite production reduce epithelial MHC-II expression and increase susceptibility to *Clostridioides difficile* infection, whereas restoration of microbiota-derived acetate rescues epithelial antigen presentation and improve disease outcome [14]. These findings suggest that epithelial MHC-II represents a physiological component of host defense that links nutritional status, microbial metabolism, and adaptive immunity during infection.

### 7.5. Epithelial MHC-II and Tumor Surveillance

Beyond its roles in mucosal homeostasis and inflammatory disease, epithelial MHC-II may contribute to immune surveillance against intestinal tumor initiation. The most direct evidence comes from studies examining the effects of a high-fat diet on intestinal stem cells [73]. High-fat feeding reduced MHC-II expression in intestinal epithelial cells, including Lgr5^+^ stem cells, in association with alterations in the intestinal microbiota. Genetic loss of epithelial MHC-II increased tumor initiation in a stem-cell-based model, supporting the concept that MHC-II-dependent interactions between epithelial cells and CD4^+^ T cells can restrain early intestinal tumorigenesis [73].

These observations are consistent with broader evidence that MHC-II expression by malignant cells can enhance antitumor immunity by enabling direct recognition by tumor-reactive CD4^+^ T cells and promoting wider immune activation [163]. However, the consequences of nonprofessional MHC-II expression are highly context-dependent. In settings where antigen presentation occurs without adequate costimulation, MHC-II-expressing tumor or stromal cells may instead induce dysfunctional effector responses or favor regulatory T-cell expansion, thereby contributing to immune tolerance and metastatic progression [164]. Thus, epithelial or tumor-cell MHC-II should not be viewed as uniformly protective, but rather as a context-dependent immune checkpoint whose outcome is shaped by antigen availability, inflammatory signaling, costimulatory capacity, and the phenotype of responding CD4^+^ T cells.

The constitutively higher expression of epithelial MHC-II in the small intestine compared with the healthy colon raises the intriguing possibility that regional differences in antigen presentation contribute to differences in tumor immune surveillance. Primary malignancies are substantially less common in the small intestine than in the colon; however, this disparity is likely determined by multiple anatomical, microbial, metabolic, and epithelial factors. At present, there is no direct evidence that regional MHC-II expression explains this difference in cancer incidence. Nevertheless, the tumor-suppressive effects observed in experimental models suggest that this hypothesis warrants further investigation, particularly through studies comparing epithelial antigen presentation and tumor-specific CD4^+^ T-cell responses across intestinal regions.

### 7.6. A Context-Dependent Regulator of Intestinal Disease

Collectively, these diverse disease models illustrate that epithelial MHC-II cannot be classified as uniformly protective or pathogenic. Instead, its biological function is highly dependent upon the surrounding tissue environment. During homeostasis, epithelial antigen presentation contributes to immune tolerance and maintenance of host–microbiota symbiosis. During acute infection, transient induction of MHC-II facilitates protective immune responses while preserving epithelial integrity. Conversely, persistent activation of the same pathway during chronic inflammatory disorders may perpetuate interactions between epithelial cells and pathogenic T-cell populations, sustaining tissue injury and barrier dysfunction.

This context dependence likely reflects the unique position occupied by intestinal epithelial cells. Unlike professional antigen-presenting cells, which primarily initiate adaptive immunity, epithelial cells continuously integrate signals derived from microbial colonization, dietary metabolites, inflammatory cytokines, and tissue damage. The outcome of antigen presentation therefore depends not simply on MHC-II itself but on the broader cellular and molecular environment in which antigen presentation occurs.

### 7.7. Therapeutic Implications

Recognition of epithelial MHC-II as a dynamic regulator of mucosal immunity raises intriguing therapeutic opportunities. Rather than broadly suppressing antigen presentation, future strategies may seek to restore physiological regulation of epithelial MHC-II by targeting upstream pathways governing epithelial–immune communication. Manipulation of the intestinal microbiota, dietary interventions that enhance beneficial microbial metabolism, cytokine modulation, or selective targeting of epithelial CIITA signaling could potentially recalibrate epithelial antigen presentation without compromising systemic immune function. Such approaches may prove particularly valuable in disorders characterized by impaired barrier homeostasis, where restoration of epithelial immune regulation may be more beneficial than generalized immunosuppression.

Although substantial progress has been made, important questions remain unanswered. It remains unclear whether disease-associated epithelial MHC-II primarily reflects altered antigen presentation, changes in epithelial lineage composition, or remodeling of local immune niches. Likewise, the identity of disease-relevant antigens presented by epithelial cells has yet to be determined. Addressing these questions will be essential for defining the therapeutic potential of epithelial antigen presentation and for understanding how disruption of this highly specialized immune pathway contributes to intestinal pathology.

## 8. Conclusions

The study of epithelial MHC-II has undergone a remarkable conceptual transformation over the past two decades. What was once considered an incidental consequence of inflammatory cytokine exposure is now recognized as a highly regulated immune program that enables intestinal epithelial cells to participate directly in adaptive immune regulation. Rather than functioning solely as a physical barrier separating the host from the luminal environment, the intestinal epithelium has emerged as an active immunological interface capable of sensing environmental cues, integrating signals from the microbiota and immune system, and communicating this information to resident CD4^+^ T cells through antigen presentation. Collectively, the evidence discussed in this review supports a revised paradigm in which epithelial MHC-II functions as a dynamic regulator of host–microbiota interactions, mucosal immune homeostasis, and barrier immunity.

Despite these advances, several important questions remain unresolved. Perhaps the most fundamental concerns the identity of the antigens presented by intestinal epithelial cells under physiological conditions. While epithelial MHC-II clearly influences bacteria-reactive T-cell populations, the relative contribution of microbial, dietary, self-derived, and pathogen-derived peptides to the epithelial immunopeptidome remains largely unknown. Recent advances in mass spectrometry-based immunopeptidomics provide unprecedented opportunities to directly characterize the peptide repertoire presented by epithelial cells in vivo and to determine how antigen specificity shapes immune tolerance, host defense, and inflammatory disease.

A second major challenge is defining the functional specialization of individual epithelial subsets. Single-cell transcriptomic studies have revealed substantial heterogeneity in epithelial MHC-II expression, yet it remains unclear which epithelial populations actively process and present antigens and how these interactions vary across distinct anatomical niches. Integration of spatial transcriptomics, multiplex imaging, lineage tracing, and organoid-based functional approaches will be essential for resolving the cellular and spatial organization of epithelial antigen presentation throughout the intestinal tract.

Another important frontier concerns the integration of environmental signals that regulate epithelial antigen presentation. As highlighted throughout this review, microbial colonization, inflammatory cytokines, dietary factors, and microbial metabolites all influence epithelial MHC-II expression and function. However, these signals operate within highly interconnected networks rather than as isolated pathways. Defining how nutritional status, microbial metabolism, immune activation, and epithelial differentiation converge to regulate antigen presentation will be critical for developing a systems-level understanding of epithelial immune regulation.

Equally important is a better understanding of the cellular consequences of epithelial antigen presentation. Current evidence suggests that intestinal epithelial cells primarily modulate tissue-resident and antigen-experienced CD4^+^ T cells rather than initiate naïve T-cell responses. However, whether epithelial MHC-II preferentially promotes immune tolerance, supports effector responses, maintains tissue-resident memory populations, or dynamically coordinates these functions according to local context remains incompletely understood. Resolving these questions will require approaches capable of simultaneously tracking antigen presentation, T-cell receptor specificity, and cellular function within intact tissue microenvironments.

The therapeutic implications of these discoveries are only beginning to emerge. Most current therapies for chronic intestinal inflammation broadly suppress immune activation. In contrast, modulation of epithelial antigen presentation may offer opportunities to restore physiological immune regulation at the mucosal interface while preserving systemic immune competence. Strategies aimed at manipulating the intestinal microbiota, enhancing beneficial microbial metabolism through dietary intervention, or targeting epithelial signaling pathways may ultimately provide novel approaches for treating IBD, enteric infection, and other disorders associated with barrier dysfunction.

In conclusion, epithelial MHC-II should no longer be viewed as a passive marker of inflammation. Instead, it represents a highly regulated and environmentally responsive pathway that enables intestinal epithelial cells to actively shape local adaptive immune responses. By integrating microbial, dietary, and immune-derived signals, epithelial antigen presentation helps coordinate tolerance, host defense, and tissue homeostasis at the mucosal surface. Continued investigation of this specialized antigen-presenting system will not only deepen our understanding of epithelial–immune communication but may also uncover new therapeutic opportunities for human disease.

## Data Availability

No new data were created or analyzed in this study. Data sharing is not applicable to this article.

## Abbreviations

The following abbreviations are used in this manuscript:

AMP	Antimicrobial peptide
APC	Antigen-presenting cell
CIITA	Class II transactivator
DCSC	Deep crypt secretory cell
ER	Endoplasmic reticulum
FFAR2	Free fatty acid receptor 2
GF	Germ-free
GVHD	Graft-versus-host disease
IBD	Inflammatory bowel disease
IEC	Intestinal epithelial cell
IEL	Intraepithelial lymphocyte
IFNγ	Interferon-γ
ILC	Innate lymphoid cell
IL	Interleukin
MHC-I	Major histocompatibility complex class I
MHC-II	Major histocompatibility complex class II
MUC2	Mucin 2
PRR	Pattern-recognition receptor
SCFA	Short-chain fatty acid
SFB	Segmented filamentous bacteria
TGF-β	Transforming growth factor-β
TLR	Toll-like receptor
Treg	Regulatory T cell
TSLP	Thymic stromal lymphopoietin

## References

[1] Artis D. (2008). Epithelial-Cell Recognition of Commensal Bacteria and Maintenance of Immune Homeostasis in the Gut. Nat. Rev. Immunol..

[2] Birchenough G.M.H., Johansson M.E., Gustafsson J.K., Bergström J.H., Hansson G.C. (2015). New Developments in Goblet Cell Mucus Secretion and Function. Mucosal Immunol..

[3] Didriksen B.J., Eshleman E.M., Alenghat T. (2024). Epithelial Regulation of Microbiota-Immune Cell Dynamics. Mucosal Immunol..

[4] Zuo L., Kuo W.-T., Turner J.R. (2020). Tight Junctions as Targets and Effectors of Mucosal Immune Homeostasis. Cell. Mol. Gastroenterol. Hepatol..

[5] Allaire J.M., Crowley S.M., Law H.T., Chang S.-Y., Ko H.-J., Vallance B.A. (2018). The Intestinal Epithelium: Central Coordinator of Mucosal Immunity. Trends Immunol..

[6] Heuberger C.E., Janney A., Ilott N., Bertocchi A., Pott S., Gu Y., Pohin M., Friedrich M., Mann E.H., Pearson C. (2024). MHC Class II Antigen Presentation by Intestinal Epithelial Cells Fine-Tunes Bacteria-Reactive CD4 T-Cell Responses. Mucosal Immunol..

[7] Koyama M., Mukhopadhyay P., Schuster I.S., Henden A.S., Hülsdünker J., Varelias A., Vetizou M., Kuns R.D., Robb R.J., Zhang P. (2019). MHC Class II Antigen Presentation by the Intestinal Epithelium Initiates Graft-versus-Host Disease and Is Influenced by the Microbiota. Immunity.

[8] Biton M., Haber A.L., Rogel N., Burgin G., Beyaz S., Schnell A., Ashenberg O., Su C.-W., Smillie C., Shekhar K. (2018). T Helper Cell Cytokines Modulate Intestinal Stem Cell Renewal and Differentiation. Cell.

[9] Stephens W.Z., Kubinak J.L., Ghazaryan A., Bauer K.M., Bell R., Buhrke K., Chiaro T.R., Weis A.M., Tang W.W., Monts J.K. (2021). Epithelial-Myeloid Exchange of MHC Class II Constrains Immunity and Microbiota Composition. Cell Rep..

[10] Van Der Kraak L.A., Schneider C., Dang V., Burr A.H.P., Weiss E.S., Varghese J.A., Yang L., Hand T.W., Canna S.W. (2021). Genetic and Commensal Induction of IL-18 Drive Intestinal Epithelial MHCII via IFNγ. Mucosal Immunol..

[11] Brabec T., Schwarzer M., Kováčová K., Dobešová M., Schierová D., Březina J., Pacáková I., Šrůtková D., Ben-Nun O., Goldfarb Y. (2024). Segmented Filamentous Bacteria–Induced Epithelial MHCII Regulates Cognate CD4^+^ IELs and Epithelial Turnover. J. Exp. Med..

[12] Thelemann C., Eren R.O., Coutaz M., Brasseit J., Bouzourene H., Rosa M., Duval A., Lavanchy C., Mack V., Mueller C. (2014). Interferon-γ Induces Expression of MHC Class II on Intestinal Epithelial Cells and Protects Mice from Colitis. PLoS ONE.

[13] Rahmani S., Galipeau H.J., Clarizio A.V., Wang X., Hann A., Rueda G.H., Kirtikar U.N., Constante M., Wulczynski M., Su H.-M. (2024). Gluten-Dependent Activation of CD4^+^ T Cells by MHC Class II–Expressing Epithelium. Gastroenterology.

[14] Fachi J.L., de Oliveira S., Trsan T., Penati S., Gilfillan S., Cao S., Ribeiro Castro P., Fernandes M.F., Hyrc K.L., Liu X. (2025). Fiber- and Acetate-Mediated Modulation of MHC-II Expression on Intestinal Epithelium Protects from *Clostridioides Difficile* Infection. Cell Host Microbe.

[15] Brown H., Esterházy D. (2021). Intestinal Immune Compartmentalization: Implications of Tissue Specific Determinants in Health and Disease. Mucosal Immunol..

[16] Mowat A.M., Agace W.W. (2014). Regional Specialization within the Intestinal Immune System. Nat. Rev. Immunol..

[17] Peterson L.W., Artis D. (2014). Intestinal Epithelial Cells: Regulators of Barrier Function and Immune Homeostasis. Nat. Rev. Immunol..

[18] Barker N. (2014). Adult Intestinal Stem Cells: Critical Drivers of Epithelial Homeostasis and Regeneration. Nat. Rev. Mol. Cell Biol..

[19] Beumer J., Clevers H. (2021). Cell Fate Specification and Differentiation in the Adult Mammalian Intestine. Nat. Rev. Mol. Cell Biol..

[20] van der Flier L.G., Clevers H. (2009). Stem Cells, Self-Renewal, and Differentiation in the Intestinal Epithelium. Annu. Rev. Physiol..

[21] Johansson M.E.V., Phillipson M., Petersson J., Velcich A., Holm L., Hansson G.C. (2008). The Inner of the Two Muc2 Mucin-Dependent Mucus Layers in Colon Is Devoid of Bacteria. Proc. Natl. Acad. Sci. USA.

[22] Kayama H., Okumura R., Takeda K. (2020). Interaction Between the Microbiota, Epithelia, and Immune Cells in the Intestine. Annu. Rev. Immunol..

[23] Mukherjee S., Zheng H., Derebe M.G., Callenberg K.M., Partch C.L., Rollins D., Propheter D.C., Rizo J., Grabe M., Jiang Q.-X. (2014). Antibacterial Membrane Attack by a Pore-Forming Intestinal C-Type Lectin. Nature.

[24] Sato T., van Es J.H., Snippert H.J., Stange D.E., Vries R.G., van den Born M., Barker N., Shroyer N.F., van de Wetering M., Clevers H. (2011). Paneth Cells Constitute the Niche for Lgr5 Stem Cells in Intestinal Crypts. Nature.

[25] Wallaeys C., Garcia-Gonzalez N., Libert C. (2023). Paneth Cells as the Cornerstones of Intestinal and Organismal Health: A Primer. EMBO Mol. Med..

[26] Rothenberg M.E., Nusse Y., Kalisky T., Lee J.J., Dalerba P., Scheeren F., Lobo N., Kulkarni S., Sim S., Qian D. (2012). Identification of a CKit^+^ Colonic Crypt Base Secretory Cell That Supports Lgr5^+^ Stem Cells in Mice. Gastroenterology.

[27] Sasaki N., Sachs N., Wiebrands K., Ellenbroek S.I.J., Fumagalli A., Lyubimova A., Begthel H., van den Born M., van Es J.H., Karthaus W.R. (2016). Reg4^+^ Deep Crypt Secretory Cells Function as Epithelial Niche for Lgr5^+^ Stem Cells in Colon. Proc. Natl. Acad. Sci. USA.

[28] Smith C.A., O’Flaherty E.A.A., Guccio N., Punnoose A., Darwish T., Lewis J.E., Foreman R.E., Li J., Kay R.G., Adriaenssens A.E. (2024). Single-Cell Transcriptomic Atlas of Enteroendocrine Cells along the Murine Gastrointestinal Tract. PLoS ONE.

[29] Gribble F.M., Reimann F. (2016). Enteroendocrine Cells: Chemosensors in the Intestinal Epithelium. Annu. Rev. Physiol..

[30] Feng X., Flüchter P., De Tenorio J.C., Schneider C. (2024). Tuft Cells in the Intestine, Immunity and Beyond. Nat. Rev. Gastroenterol. Hepatol..

[31] Mabbott N.A., Donaldson D.S., Ohno H., Williams I.R., Mahajan A. (2013). Microfold (M) Cells: Important Immunosurveillance Posts in the Intestinal Epithelium. Mucosal Immunol..

[32] Park J., Kotani T., Konno T., Setiawan J., Kitamura Y., Imada S., Usui Y., Hatano N., Shinohara M., Saito Y. (2016). Promotion of Intestinal Epithelial Cell Turnover by Commensal Bacteria: Role of Short-Chain Fatty Acids. PLoS ONE.

[33] Zheng L., Kelly C.J., Battista K.D., Schaefer R., Lanis J.M., Alexeev E.E., Wang R.X., Onyiah J.C., Kominsky D.J., Colgan S.P. (2017). Microbial-Derived Butyrate Promotes Epithelial Barrier Function through IL-10 Receptor–Dependent Repression of Claudin-2. J. Immunol..

[34] Lanis J.M., Alexeev E.E., Curtis V.F., Kitzenberg D.A., Kao D.J., Battista K.D., Gerich M.E., Glover L.E., Kominsky D.J., Colgan S.P. (2017). Tryptophan Metabolite Activation of the Aryl Hydrocarbon Receptor Regulates IL-10 Receptor Expression on Intestinal Epithelia. Mucosal Immunol..

[35] Okada T., Fukuda S., Hase K., Nishiumi S., Izumi Y., Yoshida M., Hagiwara T., Kawashima R., Yamazaki M., Oshio T. (2013). Microbiota-Derived Lactate Accelerates Colon Epithelial Cell Turnover in Starvation-Refed Mice. Nat. Commun..

[36] Rakoff-Nahoum S., Paglino J., Eslami-Varzaneh F., Edberg S., Medzhitov R. (2004). Recognition of Commensal Microflora by Toll-Like Receptors Is Required for Intestinal Homeostasis. Cell.

[37] Burgueño J.F., Abreu M.T. (2020). Epithelial Toll-like Receptors and Their Role in Gut Homeostasis and Disease. Nat. Rev. Gastroenterol. Hepatol..

[38] Guo H., Gibson S.A., Ting J.P.Y. (2020). Gut Microbiota, NLR Proteins, and Intestinal Homeostasis. J. Exp. Med..

[39] Kobayashi K.S., Chamaillard M., Ogura Y., Henegariu O., Inohara N., Nuñez G., Flavell R.A. (2005). Nod2-Dependent Regulation of Innate and Adaptive Immunity in the Intestinal Tract. Science.

[40] Lei-Leston A.C., Murphy A.G., Maloy K.J. (2017). Epithelial Cell Inflammasomes in Intestinal Immunity and Inflammation. Front. Immunol..

[41] Gourbeyre P., Berri M., Lippi Y., Meurens F., Vincent-Naulleau S., Laffitte J., Rogel-Gaillard C., Pinton P., Oswald I.P. (2015). Pattern Recognition Receptors in the Gut: Analysis of Their Expression along the Intestinal Tract and the Crypt/Villus Axis. Physiol. Rep..

[42] Sheridan B.S., Lefrançois L. (2010). Intraepithelial Lymphocytes: To Serve and Protect. Curr. Gastroenterol. Rep..

[43] Cheroutre H., Lambolez F., Mucida D. (2011). The Light and Dark Sides of Intestinal Intraepithelial Lymphocytes. Nat. Rev. Immunol..

[44] Malamut G., El Machhour R., Montcuquet N., Martin-Lannerée S., Dusanter-Fourt I., Verkarre V., Mention J.-J., Rahmi G., Kiyono H., Butz E.A. (2010). IL-15 Triggers an Antiapoptotic Pathway in Human Intraepithelial Lymphocytes That Is a Potential New Target in Celiac Disease–Associated Inflammation and Lymphomagenesis. J. Clin. Investig..

[45] Kennedy M.K., Glaccum M., Brown S.N., Butz E.A., Viney J.L., Embers M., Matsuki N., Charrier K., Sedger L., Willis C.R. (2000). Reversible Defects in Natural Killer and Memory CD8 T Cell Lineages in Interleukin 15-Deficient Mice. J. Exp. Med..

[46] Taylor B.C., Zaph C., Troy A.E., Du Y., Guild K.J., Comeau M.R., Artis D. (2009). TSLP Regulates Intestinal Immunity and Inflammation in Mouse Models of Helminth Infection and Colitis. J. Exp. Med..

[47] Zeuthen L.H., Fink L.N., Frokiaer H. (2008). Epithelial Cells Prime the Immune Response to an Array of Gut-derived Commensals towards a Tolerogenic Phenotype through Distinct Actions of Thymic Stromal Lymphopoietin and Transforming Growth Factor-β. Immunology.

[48] Holani R., Babbar A., Blyth G.A.D., Lopes F., Jijon H., McKay D.M., Hollenberg M.D., Cobo E.R. (2020). Cathelicidin-Mediated Lipopolysaccharide Signaling via Intracellular TLR4 in Colonic Epithelial Cells Evokes CXCL8 Production. Gut Microbes.

[49] Rimoldi M., Chieppa M., Salucci V., Avogadri F., Sonzogni A., Sampietro G.M., Nespoli A., Viale G., Allavena P., Rescigno M. (2005). Intestinal Immune Homeostasis Is Regulated by the Crosstalk between Epithelial Cells and Dendritic Cells. Nat. Immunol..

[50] Wurbel M.A., Malissen M., Guy-Grand D., Meffre E., Nussenzweig M.C., Richelme M., Carrier A., Malissen B. (2001). Mice Lacking the CCR9 CC-Chemokine Receptor Show a Mild Impairment of Early T- and B-Cell Development and a Reduction in T-Cell Receptor γδ^+^ Gut Intraepithelial Lymphocytes. Blood.

[51] Lindemans C.A., Calafiore M., Mertelsmann A.M., O’Connor M.H., Dudakov J.A., Jenq R.R., Velardi E., Young L.F., Smith O.M., Lawrence G. (2015). Interleukin-22 Promotes Intestinal-Stem-Cell-Mediated Epithelial Regeneration. Nature.

[52] Kinnebrew M.A., Buffie C.G., Diehl G.E., Zenewicz L.A., Leiner I., Hohl T.M., Flavell R.A., Littman D.R., Pamer E.G. (2012). Interleukin 23 Production by Intestinal CD103^+^CD11b^+^ Dendritic Cells in Response to Bacterial Flagellin Enhances Mucosal Innate Immune Defense. Immunity.

[53] Marillier R.G., Michels C., Smith E.M., Fick L.C., Leeto M., Dewals B., Horsnell W.G., Brombacher F. (2008). IL-4/IL-13 Independent Goblet Cell Hyperplasia in Experimental Helminth Infections. BMC Immunol..

[54] Macho-Fernandez E., Koroleva E.P., Spencer C.M., Tighe M., Torrado E., Cooper A.M., Fu Y.-X., Tumanov A.V. (2015). Lymphotoxin Beta Receptor Signaling Limits Mucosal Damage through Driving IL-23 Production by Epithelial Cells. Mucosal Immunol..

[55] Heuberger C., Pott J., Maloy K.J. (2021). Why Do Intestinal Epithelial Cells Express MHC Class II?. Immunology.

[56] Roland M.M., Mohammed A.D., Kubinak J.L. (2020). How MHCII Signaling Promotes Benign Host-Microbiota Interactions. PLoS Pathog..

[57] Roche P.A., Furuta K. (2015). The Ins and Outs of MHC Class II-Mediated Antigen Processing and Presentation. Nat. Rev. Immunol..

[58] Blum J.S., Wearsch P.A., Cresswell P. (2013). Pathways of Antigen Processing. Annu. Rev. Immunol..

[59] Selby W.S., Janossy G., Mason D.Y., Jewell D.P. (1983). Expression of HLA-DR Antigens by Colonic Epithelium in Inflammatory Bowel Disease. Clin. Exp. Immunol..

[60] Mayer L., Eisenhardt D., Salomon P., Bauer W., Plous R., Piccinini L. (1991). Expression of Class II Molecules on Intestinal Epithelial Cells in Humans. Differences between Normal and Inflammatory Bowel Disease. Gastroenterology.

[61] Hershberg R.M., Cho D.H., Youakim A., Bradley M.B., Lee J.S., Framson P.E., Nepom G.T. (1998). Highly Polarized HLA Class II Antigen Processing and Presentation by Human Intestinal Epithelial Cells. J. Clin. Investig..

[62] Wosen J.E., Mukhopadhyay D., Macaubas C., Mellins E.D. (2018). Epithelial MHC Class II Expression and Its Role in Antigen Presentation in the Gastrointestinal and Respiratory Tracts. Front. Immunol..

[63] Durai V., Murphy K.M. (2016). Functions of Murine Dendritic Cells. Immunity.

[64] Münz C. (2016). Autophagy Beyond Intracellular MHC Class II Antigen Presentation. Trends Immunol..

[65] Kulkarni D.H., Gustafsson J.K., Knoop K.A., McDonald K.G., Bidani S.S., Davis J.E., Floyd A.N., Hogan S.P., Hsieh C.-S., Newberry R.D. (2020). Goblet Cell Associated Antigen Passages Support the Induction and Maintenance of Oral Tolerance. Mucosal Immunol..

[66] Rios D., Wood M.B., Li J., Chassaing B., Gewirtz A.T., Williams I.R. (2016). Antigen Sampling by Intestinal M Cells Is the Principal Pathway Initiating Mucosal IgA Production to Commensal Enteric Bacteria. Mucosal Immunol..

[67] Hershberg R.M., Mayer L.F. (2000). Antigen Processing and Presentation by Intestinal Epithelial Cells—Polarity and Complexity. Immunol. Today.

[68] Bowcutt R. (2014). Heterogeneity across the Murine Small and Large Intestine. World J. Gastroenterol..

[69] Piskurich J.F., Linhoff M.W., Wang Y., Ting J.P.-Y. (1999). Two Distinct Gamma Interferon-Inducible Promoters of the Major Histocompatibility Complex Class II Transactivator Gene Are Differentially Regulated by STAT1, Interferon Regulatory Factor 1, and Transforming Growth Factor β. Mol. Cell. Biol..

[70] Bland P.W., Warren L.G. (1986). Antigen Presentation by Epithelial Cells of the Rat Small Intestine. II. Selective Induction of Suppressor T Cells. Immunology.

[71] Scott H., Solheim B.G., Brandtzaeg P., Thorsby E. (1980). HLA-DR-like Antigens in the Epithelium of the Human Small Intestine. Scand. J. Immunol..

[72] Haber A.L., Biton M., Rogel N., Herbst R.H., Shekhar K., Smillie C., Burgin G., Delorey T.M., Howitt M.R., Katz Y. (2017). A Single-Cell Survey of the Small Intestinal Epithelium. Nature.

[73] Beyaz S., Chung C., Mou H., Bauer-Rowe K.E., Xifaras M.E., Ergin I., Dohnalova L., Biton M., Shekhar K., Eskiocak O. (2021). Dietary Suppression of MHC Class II Expression in Intestinal Epithelial Cells Enhances Intestinal Tumorigenesis. Cell Stem Cell.

[74] Satija R., Farrell J.A., Gennert D., Schier A.F., Regev A. (2015). Spatial Reconstruction of Single-Cell Gene Expression Data. Nat. Biotechnol..

[75] Pickles O.J., Wanigasooriya K., Ptasinska A., Patel A.J., Robbins H.L., Bryer C., Whalley C.M., Tee L., Lal N., Pinna C.M.A. (2023). MHC Class II Is Induced by IFNγ and Follows Three Distinct Patterns of Expression in Colorectal Cancer Organoids. Cancer Res. Commun..

[76] León Machado J.A., Steimle V. (2021). The MHC Class II Transactivator CIITA: Not (Quite) the Odd-One-Out Anymore among NLR Proteins. Int. J. Mol. Sci..

[77] Masternak K., Muhlethaler-Mottet A., Villard J., Zufferey M., Steimle V., Reith W. (2000). CIITA Is a Transcriptional Coactivator That Is Recruited to MHC Class II Promoters by Multiple Synergistic Interactions with an Enhanceosome Complex. Genes Dev..

[78] Wong D., Lee W., Humburg P., Makino S., Lau E., Naranbhai V., Fairfax B.P., Chan K., Plant K., Knight J.C. (2014). Genomic Mapping of the MHC Transactivator CIITA Using an Integrated ChIP-Seq and Genetical Genomics Approach. Genome Biol..

[79] Reith W., LeibundGut-Landmann S., Waldburger J.-M. (2005). Regulation of MHC Class II Gene Expression by the Class II Transactivator. Nat. Rev. Immunol..

[80] Ting J.P.-Y., Trowsdale J. (2002). Genetic Control of MHC Class II Expression. Cell.

[81] Parveen S., Fatma M., Mir S., Dermime S., Uddin S. (2025). JAK-STAT Signaling in Autoimmunity and Cancer. Immunotargets Ther..

[82] Liu X., Ye L., Bai Y., Mojidi H., Simister N.E., Zhu X. (2008). Activation of the JAK/STAT-1 Signaling Pathway by IFN-Gamma Can down-Regulate Functional Expression of the MHC Class I-Related Neonatal Fc Receptor for IgG. J. Immunol..

[83] Rosowski E.E., Saeij J.P.J. (2012). Toxoplasma Gondii Clonal Strains All Inhibit STAT1 Transcriptional Activity but Polymorphic Effectors Differentially Modulate IFNγ Induced Gene Expression and STAT1 Phosphorylation. PLoS ONE.

[84] Hardy P., Diallo T.O., Matte C., Descoteaux A. (2009). Roles of Phosphatidylinositol 3-kinase and P38 Mitogen-activated Protein Kinase in the Regulation of Protein Kinase C-α Activation in Interferon-γ-stimulated Macrophages. Immunology.

[85] Chen L., Jiang H., Tan B., Geng R., Chen J., Wu S., Fang Z., Lang Y., Ma H., Zheng X. (2025). IL-1β-Mediated Suppression of CIITA Attenuates IFN-γ-Induced MHC-II Expression on Fibroblasts. Cell Commun. Signal..

[86] Gopalakrishnan S., Hansen M.D., Skovdahl H.K., Roseth I.A., van Beelen Granlund A., Østvik A.E., Bakke I., Sandvik A.K., Bruland T. (2022). Tofacitinib Downregulates TNF and Poly(I:C)-Dependent MHC-II Expression in the Colonic Epithelium. Front. Immunol..

[87] Hu Q., Bian Q., Rong D., Wang L., Song J., Huang H.-S., Zeng J., Mei J., Wang P.-Y. (2023). JAK/STAT Pathway: Extracellular Signals, Diseases, Immunity, and Therapeutic Regimens. Front. Bioeng. Biotechnol..

[88] Eshleman E.M., Shao T.-Y., Woo V., Rice T., Engleman L., Didriksen B.J., Whitt J., Haslam D.B., Way S.S., Alenghat T. (2023). Intestinal Epithelial HDAC3 and MHC Class II Coordinate Microbiota-Specific Immunity. J. Clin. Investig..

[89] Lebrusant-Fernandez M., ap Rees T., Jimeno R., Angelis N., Ng J.C., Fraternali F., Li V.S.W., Barral P. (2024). IFN-γ-Dependent Regulation of Intestinal Epithelial Homeostasis by NKT Cells. Cell Rep..

[90] MacDonald T.T., Weinel A., Spencer J. (1988). HLA-DR Expression in Human Fetal Intestinal Epithelium. Gut.

[91] Lin X.P., Almqvist N., Telemo E. (2005). Human Small Intestinal Epithelial Cells Constitutively Express the Key Elements for Antigen Processing and the Production of Exosomes. Blood Cells Mol. Dis..

[92] Chiba M., Iizuka M., Masamune O. (1988). Ubiquitous Expression of HLA-DR Antigens on Human Small Intestinal Epithelium. Gastroenterol. Jpn..

[93] Koretz K., Momburg F., Otto H.F., Möller P. (1987). Sequential Induction of MHC Antigens on Autochthonous Cells of Ileum Affected by Crohn’s Disease. Am. J. Pathol..

[94] Mason D.W., Dallman M., Barclay A.N. (1981). Graft-versus-Host Disease Induces Expression of Ia Antigen in Rat Epidermal Cells and Gut Epithelium. Nature.

[95] Arnaud-Battandier F., Cerf-Bensussan N., Amsellem R., Schmitz J. (1986). Increased HLA-DR Expression by Enterocytes in Children with Celiac Disease. Gastroenterology.

[96] Dotan I., Allez M., Nakazawa A., Brimnes J., Schulder-Katz M., Mayer L. (2007). Intestinal Epithelial Cells from Inflammatory Bowel Disease Patients Preferentially Stimulate CD4^+^ T Cells to Proliferate and Secrete Interferon-γ. Am. J. Physiol.-Gastrointest. Liver Physiol..

[97] Fais S., Maiuri L., Pallone F., De Vincenzi M., De Ritis G., Troncone R., Auricchio S. (1992). Gliadin Induced Changes in the Expression of MHC-Class II Antigens by Human Small Intestinal Epithelium. Organ Culture Studies with Coeliac Disease Mucosa. Gut.

[98] Chougule P., Herlenius G., Hernandez N.M., Patil P.B., Xu B., Sumitran-Holgersson S. (2012). Isolation and Characterization of Human Primary Enterocytes from Small Intestine Using a Novel Method. Scand. J. Gastroenterol..

[99] Ghilas S., O’Keefe R., Mielke L.A., Raghu D., Buchert M., Ernst M. (2022). Crosstalk between Epithelium, Myeloid and Innate Lymphoid Cells during Gut Homeostasis and Disease. Front. Immunol..

[100] Omrani O., Krepelova A., Rasa S.M.M., Sirvinskas D., Lu J., Annunziata F., Garside G., Bajwa S., Reinhardt S., Adam L. (2023). IFNγ-Stat1 Axis Drives Aging-Associated Loss of Intestinal Tissue Homeostasis and Regeneration. Nat. Commun..

[101] Osborn J., Greer S. (2015). Metastatic Melanoma Cells Evade Immune Detection by Silencing STAT1. Int. J. Mol. Sci..

[102] Koyama M., Hippe D.S., Srinivasan S., Proll S.C., Miltiadous O., Li N., Zhang P., Ensbey K.S., Hoffman N.G., Schmidt C.R. (2023). Intestinal Microbiota Controls Graft-versus-Host Disease Independent of Donor-Host Genetic Disparity. Immunity.

[103] Moon S., Park Y., Hyeon S., Kim Y.-M., Kim J.-H., Kim H., Park S., Lee K.-J., Koo B.-K., Ha S.-J. (2021). Niche-Specific MHC II and PD-L1 Regulate CD4^+^CD8αα^+^ Intraepithelial Lymphocyte Differentiation. J. Exp. Med..

[104] Hershberg R.M., Framson P.E., Cho D.H., Lee L.Y., Kovats S., Beitz J., Blum J.S., Nepom G.T. (1997). Intestinal Epithelial Cells Use Two Distinct Pathways for HLA Class II Antigen Processing. J. Clin. Investig..

[105] Nava P., Koch S., Laukoetter M.G., Lee W.Y., Kolegraff K., Capaldo C.T., Beeman N., Addis C., Gerner-Smidt K., Neumaier I. (2010). Interferon-γ Regulates Intestinal Epithelial Homeostasis through Converging β-Catenin Signaling Pathways. Immunity.

[106] Rodriguez-Marino N., Royer C.J., Rivera-Rodriguez D.E., Seto E., Gracien I., Jones R.M., Scharer C.D., Gracz A.D., Cervantes-Barragan L. (2024). Dietary Fiber Promotes Antigen Presentation on Intestinal Epithelial Cells and Development of Small Intestinal CD4^+^CD8αα^+^ Intraepithelial T Cells. Mucosal Immunol..

[107] Van Kaer L., Olivares-Villagómez D. (2018). Development, Homeostasis, and Functions of Intestinal Intraepithelial Lymphocytes. J. Immunol..

[108] Lockhart A., Mucida D., Bilate A.M. (2024). Intraepithelial Lymphocytes of the Intestine. Annu. Rev. Immunol..

[109] Saez A., Gomez-Bris R., Herrero-Fernandez B., Mingorance C., Rius C., Gonzalez-Granado J.M. (2021). Innate Lymphoid Cells in Intestinal Homeostasis and Inflammatory Bowel Disease. Int. J. Mol. Sci..

[110] Vivier E., Artis D., Colonna M., Diefenbach A., Di Santo J.P., Eberl G., Koyasu S., Locksley R.M., McKenzie A.N.J., Mebius R.E. (2018). Innate Lymphoid Cells: 10 Years On. Cell.

[111] Marrero I., Maricic I., Feldstein A.E., Loomba R., Schnabl B., Rivera-Nieves J., Eckmann L., Kumar V. (2018). Complex Network of NKT Cell Subsets Controls Immune Homeostasis in Liver and Gut. Front. Immunol..

[112] Sullivan D.P., Bui T., Muller W.A., Butin-Israeli V., Sumagin R. (2018). In Vivo Imaging Reveals Unique Neutrophil Transendothelial Migration Patterns in Inflamed Intestines. Mucosal Immunol..

[113] Chou C., Li M.O. (2018). Tissue-Resident Lymphocytes Across Innate and Adaptive Lineages. Front. Immunol..

[114] Pereira M.V.A., Galvani R.G., Gonçalves-Silva T., de Vasconcelo Z.F.M., Bonomo A. (2024). Tissue Adaptation of CD4 T Lymphocytes in Homeostasis and Cancer. Front. Immunol..

[115] Malik A., Sharma D., Aguirre-Gamboa R., McGrath S., Zabala S., Weber C., Jabri B. (2023). Epithelial IFNγ Signalling and Compartmentalized Antigen Presentation Orchestrate Gut Immunity. Nature.

[116] Bär F., Sina C., Hundorfean G., Pagel R., Lehnert H., Fellermann K., Büning J. (2013). Inflammatory Bowel Diseases Influence Major Histocompatibility Complex Class I (MHC I) and II Compartments in Intestinal Epithelial Cells. Clin. Exp. Immunol..

[117] Tindemans I., Joosse M.E., Samsom J.N. (2020). Dissecting the Heterogeneity in T-Cell Mediated Inflammation in IBD. Cells.

[118] Wang Z., Wang Z., Wang J., Diao Y., Qian X., Zhu N. (2016). T-Bet-Expressing B Cells Are Positively Associated with Crohn’s Disease Activity and Support Th1 Inflammation. DNA Cell Biol..

[119] Landy E., Carol H., Ring A., Canna S. (2024). Biological and Clinical Roles of IL-18 in Inflammatory Diseases. Nat. Rev. Rheumatol..

[120] Allam O., Samarani S., Mehraj V., Jenabian M.-A., Tremblay C., Routy J.-P., Amre D., Ahmad A. (2018). HIV Induces Production of IL-18 from Intestinal Epithelial Cells That Increases Intestinal Permeability and Microbial Translocation. PLoS ONE.

[121] Nakanishi K. (2018). Unique Action of Interleukin-18 on T Cells and Other Immune Cells. Front. Immunol..

[122] Gullicksrud J.A., Sateriale A., Engiles J.B., Gibson A.R., Shaw S., Hutchins Z.A., Martin L., Christian D.A., Taylor G.A., Yamamoto M. (2022). Enterocyte–Innate Lymphoid Cell Crosstalk Drives Early IFN-γ-Mediated Control of Cryptosporidium. Mucosal Immunol..

[123] Kak G., Raza M., Tiwari B.K. (2018). Interferon-Gamma (IFN-γ): Exploring Its Implications in Infectious Diseases. Biomol. Concepts.

[124] Kominsky D.J., Campbell E.L., Ehrentraut S.F., Wilson K.E., Kelly C.J., Glover L.E., Collins C.B., Bayless A.J., Saeedi B., Dobrinskikh E. (2014). IFN-γ–Mediated Induction of an Apical IL-10 Receptor on Polarized Intestinal Epithelia. J. Immunol..

[125] Dudakov J.A., Hanash A.M., van den Brink M.R.M. (2015). Interleukin-22: Immunobiology and Pathology. Annu. Rev. Immunol..

[126] Patnaude L., Mayo M., Mario R., Wu X., Knight H., Creamer K., Wilson S., Pivorunas V., Karman J., Phillips L. (2021). Mechanisms and Regulation of IL-22-Mediated Intestinal Epithelial Homeostasis and Repair. Life Sci..

[127] Moniruzzaman M., Rahman M.A., Wang R., Wong K.Y., Chen A.C.-H., Mueller A., Taylor S., Harding A., Illankoon T., Wiid P. (2023). Interleukin-22 Suppresses Major Histocompatibility Complex II in Mucosal Epithelial Cells. J. Exp. Med..

[128] Pennino D., Bhavsar P.K., Effner R., Avitabile S., Venn P., Quaranta M., Marzaioli V., Cifuentes L., Durham S.R., Cavani A. (2013). IL-22 Suppresses IFN-γ–Mediated Lung Inflammation in Asthmatic Patients. J. Allergy Clin. Immunol..

[129] Balmer M.L., Ma E.H., Thompson A.J., Epple R., Unterstab G., Lötscher J., Dehio P., Schürch C.M., Warncke J.D., Perrin G. (2020). Memory CD8^+^ T Cells Balance Pro- and Anti-Inflammatory Activity by Reprogramming Cellular Acetate Handling at Sites of Infection. Cell Metab..

[130] Niu J., Cui M., Yang X., Li J., Yao Y., Guo Q., Lu A., Qi X., Zhou D., Zhang C. (2023). Microbiota-Derived Acetate Enhances Host Antiviral Response via NLRP3. Nat. Commun..

[131] Qiu J., Villa M., Sanin D.E., Buck M.D., O’Sullivan D., Ching R., Matsushita M., Grzes K.M., Winkler F., Chang C.-H. (2019). Acetate Promotes T Cell Effector Function during Glucose Restriction. Cell Rep..

[132] Chun E., Lavoie S., Fonseca-Pereira D., Bae S., Michaud M., Hoveyda H.R., Fraser G.L., Gallini Comeau C.A., Glickman J.N., Fuller M.H. (2019). Metabolite-Sensing Receptor Ffar2 Regulates Colonic Group 3 Innate Lymphoid Cells and Gut Immunity. Immunity.

[133] Fachi J.L., Sécca C., Rodrigues P.B., de Mato F.C.P., Di Luccia B., de Souza F.J., Pral L.P., Rungue M., de Melo R.V., Sato F.T. (2020). Acetate Coordinates Neutrophil and ILC3 Responses against *C. Difficile* through FFAR2. J. Exp. Med..

[134] Belkaid Y., Harrison O.J. (2017). Homeostatic Immunity and the Microbiota. Immunity.

[135] Matsumoto S., Setoyama H., Umesaki Y. (1992). Differential Induction of Major Histocompatibility Complex Molecules on Mouse Intestine by Bacterial Colonization. Gastroenterology.

[136] Rask C., Evertsson S., Telemo E., Wold A.E. (2005). A Full Flora, but Not Monocolonization by Escherichia Coli or Lactobacilli, Supports Tolerogenic Processing of a Fed Antigen. Scand. J. Immunol..

[137] Reikvam D.H., Erofeev A., Sandvik A., Grcic V., Jahnsen F.L., Gaustad P., McCoy K.D., Macpherson A.J., Meza-Zepeda L.A., Johansen F.-E. (2011). Depletion of Murine Intestinal Microbiota: Effects on Gut Mucosa and Epithelial Gene Expression. PLoS ONE.

[138] Chen B., Ni X., Sun R., Zeng B., Wei H., Tian Z., Wei H. (2018). Commensal Bacteria-Dependent CD8αβ^+^ T Cells in the Intestinal Epithelium Produce Antimicrobial Peptides. Front. Immunol..

[139] Han G., Luong H., Vaishnava S. (2022). Low Abundance Members of the Gut Microbiome Exhibit High Immunogenicity. Gut Microbes.

[140] Carroll S.L., Pasare C., Barton G.M. (2024). Control of Adaptive Immunity by Pattern Recognition Receptors. Immunity.

[141] Abreu M.T. (2010). Toll-like Receptor Signalling in the Intestinal Epithelium: How Bacterial Recognition Shapes Intestinal Function. Nat. Rev. Immunol..

[142] Frantz A.L., Rogier E.W., Weber C.R., Shen L., Cohen D.A., Fenton L.A., Bruno M.E.C., Kaetzel C.S. (2012). Targeted Deletion of MyD88 in Intestinal Epithelial Cells Results in Compromised Antibacterial Immunity Associated with Downregulation of Polymeric Immunoglobulin Receptor, Mucin-2, and Antibacterial Peptides. Mucosal Immunol..

[143] Ivanov I.I., Atarashi K., Manel N., Brodie E.L., Shima T., Karaoz U., Wei D., Goldfarb K.C., Santee C.A., Lynch S.V. (2009). Induction of Intestinal Th17 Cells by Segmented Filamentous Bacteria. Cell.

[144] Lécuyer E., Rakotobe S., Lengliné-Garnier H., Lebreton C., Picard M., Juste C., Fritzen R., Eberl G., McCoy K.D., Macpherson A.J. (2014). Segmented Filamentous Bacterium Uses Secondary and Tertiary Lymphoid Tissues to Induce Gut IgA and Specific T Helper 17 Cell Responses. Immunity.

[145] Round J.L., Mazmanian S.K. (2009). The Gut Microbiota Shapes Intestinal Immune Responses during Health and Disease. Nat. Rev. Immunol..

[146] Thaiss C.A., Zmora N., Levy M., Elinav E. (2016). The Microbiome and Innate Immunity. Nature.

[147] Smith P.M., Howitt M.R., Panikov N., Michaud M., Gallini C.A., Bohlooly Y.M., Glickman J.N., Garrett W.S. (2013). The Microbial Metabolites, Short-Chain Fatty Acids, Regulate Colonic Treg Cell Homeostasis. Science.

[148] Alenghat T., Artis D. (2014). Epigenomic Regulation of Host–Microbiota Interactions. Trends Immunol..

[149] Hills R., Pontefract B., Mishcon H., Black C., Sutton S., Theberge C. (2019). Gut Microbiome: Profound Implications for Diet and Disease. Nutrients.

[150] Koh A., De Vadder F., Kovatcheva-Datchary P., Bäckhed F. (2016). From Dietary Fiber to Host Physiology: Short-Chain Fatty Acids as Key Bacterial Metabolites. Cell.

[151] Corrêa-Oliveira R., Fachi J.L., Vieira A., Sato F.T., Vinolo M.A.R. (2016). Regulation of Immune Cell Function by Short-Chain Fatty Acids. Clin. Transl. Immunol..

[152] Postler T.S., Ghosh S. (2017). Understanding the Holobiont: How Microbial Metabolites Affect Human Health and Shape the Immune System. Cell Metab..

[153] Zelante T., Iannitti R.G., Cunha C., De Luca A., Giovannini G., Pieraccini G., Zecchi R., D’Angelo C., Massi-Benedetti C., Fallarino F. (2013). Tryptophan Catabolites from Microbiota Engage Aryl Hydrocarbon Receptor and Balance Mucosal Reactivity via Interleukin-22. Immunity.

[154] Sonnenburg E.D., Smits S.A., Tikhonov M., Higginbottom S.K., Wingreen N.S., Sonnenburg J.L. (2016). Diet-Induced Extinctions in the Gut Microbiota Compound over Generations. Nature.

[155] Makki K., Deehan E.C., Walter J., Bäckhed F. (2018). The Impact of Dietary Fiber on Gut Microbiota in Host Health and Disease. Cell Host Microbe.

[156] Jamwal D.R., Laubitz D., Harrison C.A., Figliuolo da Paz V., Cox C.M., Wong R., Midura-Kiela M., Gurney M.A., Besselsen D.G., Setty P. (2020). Intestinal Epithelial Expression of MHCII Determines Severity of Chemical, T-Cell–Induced, and Infectious Colitis in Mice. Gastroenterology.

[157] Sujino T., London M., Hoytema van Konijnenburg D.P., Rendon T., Buch T., Silva H.M., Lafaille J.J., Reis B.S., Mucida D. (2016). Tissue Adaptation of Regulatory and Intraepithelial CD4^+^ T Cells Controls Gut Inflammation. Science.

[158] Chang J.T. (2020). Pathophysiology of Inflammatory Bowel Diseases. N. Engl. J. Med..

[159] Jabri B., Sollid L.M. (2017). T Cells in Celiac Disease. J. Immunol..

[160] Abadie V., Jabri B. (2014). IL-15: A Central Regulator of Celiac Disease Immunopathology. Immunol. Rev..

[161] Sollid L.M., Jabri B. (2013). Triggers and Drivers of Autoimmunity: Lessons from Coeliac Disease. Nat. Rev. Immunol..

[162] Zeiser R., Blazar B.R. (2017). Acute Graft-versus-Host Disease—Biologic Process, Prevention, and Therapy. N. Engl. J. Med..

[163] Axelrod M.L., Cook R.S., Johnson D.B., Balko J.M. (2019). Biological Consequences of MHC-II Expression by Tumor Cells in Cancer. Clin. Cancer Res..

[164] Lei P.-J., Pereira E.R., Andersson P., Amoozgar Z., Van Wijnbergen J.W., O’Melia M.J., Zhou H., Chatterjee S., Ho W.W., Posada J.M. (2023). Cancer Cell Plasticity and MHC-II-Mediated Immune Tolerance Promote Breast Cancer Metastasis to Lymph Nodes. J. Exp. Med..

